# Supplementary material for: Medicinal plants for allergic rhinitis: A systematic review and meta-analysis
Source: PLoS One. 2024 Apr 11;19(4):e0297839. doi: 10.1371/journal.pone.0297839 (PMC11008904; doi:10.1371/journal.pone.0297839)
Supplement: S1 Appendix — (DOCX) [file pone.0297839.s001.docx]

**Appendix S1: Example full search strategy**

Date of search: 18^th^ July 2023

Database: MEDLINE

**Keyword Search**

(("rhinitis, allergic"[MeSH Terms] OR ("rhinitis"[All Fields] AND "allergic"[All Fields]) OR "allergic rhinitis"[All Fields] OR ("allergic"[All Fields] AND "rhinitis"[All Fields])) AND ("herb*"[All Fields] OR "phytotherap*"[All Fields] OR "plant*"[All Fields])) AND (clinicaltrial[Filter])

**Translations**

**allergic rhinitis:** "rhinitis, allergic"[MeSH Terms] OR ("rhinitis"[All Fields] AND "allergic"[All Fields]) OR "allergic rhinitis"[All Fields] OR ("allergic"[All Fields] AND "rhinitis"[All Fields])

**MeSH Terms:**

- Rhinitis, Allergic, Nonseasonal
- Seasonal Allergic Rhinitis
- Allergic Rhinitides, Seasonal
- Allergic Rhinitis, Seasonal
- Rhinitides, Seasonal Allergic
- Rhinitis, Seasonal Allergic
- Seasonal Allergic Rhinitides
- Pollen Allergy
- Allergies, Pollen
- Allergy, Pollen
- Pollen Allergies
- Pollinosis
- Pollinoses
- Hay Fever
- Fever, Hay
- Hayfever
