# Supplementary material for: Medicinal plants for allergic rhinitis: A systematic review and meta-analysis
Source: PLoS One. 2024 Apr 11;19(4):e0297839. doi: 10.1371/journal.pone.0297839 (PMC11008904; doi:10.1371/journal.pone.0297839)
Supplement: S2 Appendix — (DOCX) [file pone.0297839.s002.docx]

**Appendix S2: Data extraction table**

|  | **Study Demographics** | | | | | | | |
| --- | --- | --- | --- | --- | --- | --- | --- | --- |
| **No** | **Author** | **Title** | **Year** | **Country** | **Study Design** | **Objectives** | **Trial Registry (Yes/Not mentioned; if yes, please mention number)** | **Ethics Approval (Yes/Not mentioned; if yes, please mention number)** |

| **Population** | | | | | | |
| --- | --- | --- | --- | --- | --- | --- |
| **Participant description** | **Inclusion criteria** | **Exclusion criteria** | **Diagnosis: Allergic Rhinitis/Allergic Rhinosinusitis/Allergic Rhinoconjunctivitis** | **Category of AR (Seasonal, Perennial, Not Mentioned)** | **Severity of AR (Mild, Moderate, Severe, Persistent, Intermittent, Not mentioned)** | **Diagnosis criteria used for AR diagnosis (If not mentioned, please state not mentioned)** |

| **Population** | | | | | | | | | |
| --- | --- | --- | --- | --- | --- | --- | --- | --- | --- |
| **Age (Mean; SD)** | **Sex (Percentage)** | **Sample size (Initial number)** | **Sample size (final number that completed study and analysed)** | **Drop out (if any)** | **Reason for drop out** | **Co-morbidities (Yes/No/ Not reported; if yes please specify)** | **Sample size calculation (Yes/Not reported; please describer if yes)** | **Power of study (Yes/ Not reported; please describer if yes)** | **Informed consent** |

| **Intervention** | | | | | | | |
| --- | --- | --- | --- | --- | --- | --- | --- |
| **Description** | **Plant Name** | **Plant part** | **Form: Fresh/ Dried (or not mentioned)** | **Formulation (e.g., capsule/decoction/extract- please mention details of extract)** | **Content of Formulation** | **Qualitative analysis of content (Yes, No; describe method used if Yes e.g. hplc/uv/tlc)** | **Quantitative analysis of content (Yes, No; describe method used if Yes e.g. hplc/uv/tlc)** |

| **Intervention** | | | | | | |
| --- | --- | --- | --- | --- | --- | --- |
| **Standardisation details (Yes/Not mentioned; describe if yes/NA)** | **Biomarker/ chemical marker (Name, quantity OR not mentioned)** | **Source (of plant/ company/ manufacturer)** | **Voucher specimen deposited? (Yes/No/Unclear) If Yes, specify details** | **Dose & Frequency** | **Duration (Days)** | **Co-intervention** |

| **Comparator** | | | |
| --- | --- | --- | --- |
| **Treatment Description** | **Dose & Frequency** | **Duration (Days)** | **Co-intervention** |

| **Outcome (Efficacy)** | | | | |
| --- | --- | --- | --- | --- |
| **Description of parameters (E.g., Daytime symptom score, Ophthalmic symptom score)** | **Unit** | **Definition** | **Methods of outcome measured (e.g., observed by practitioner/nurse/telephone calls etc)** | **Timepoint of measures taken (e.g., on day 1, 4, 6 etc.)** |

| **Outcome (Efficacy)- Intervention Group** | | | | | | | | |
| --- | --- | --- | --- | --- | --- | --- | --- | --- |
| **Endpoint** | **No of participants intervention** | **Pre intervention/ Baseline (mean ± SD) If other than mean ± SD please specify** | **Post intervention Time point 1 (e.g., day 3 mean ± SD)** | **Post intervention Time point 2** | **Post intervention Time point 3** | **Post intervention Time point 4** | **Post intervention Time point 5** | **Post intervention Time point 6** |

| **Outcome (Efficacy)- Control Group** | | | | | | | |
| --- | --- | --- | --- | --- | --- | --- | --- |
| **No of participants in control** | **Baseline in control** | **Post in control time point 1 (e.g., at day 3 mean ± SD)** | **Post in control time point 2** | **Post in control time point 3** | **Post in control time point 4** | **Post in control time point 5** | **Post in control time point 6** |

| **Outcome (Safety)** | | | |
| --- | --- | --- | --- |
| **Any adverse reaction reported? (Yes/ No- no ADR reported/ Not mentioned- totally no mention on monitoring of safety)** | **Methods of assessment (e.g., patient self-report/ assessor monitor/ lab investigations/ not mentioned)** | **Intervention group (Description and incidence)** | **Control group (Description and incidence)** |

| **Others** | | | | |
| --- | --- | --- | --- | --- |
| **Adherence monitoring** | **Limitations** | **Conclusion** | **Funding** | Comments |
