# Supplementary material for: Medicinal plants for allergic rhinitis: A systematic review and meta-analysis
Source: PLoS One. 2024 Apr 11;19(4):e0297839. doi: 10.1371/journal.pone.0297839 (PMC11008904; doi:10.1371/journal.pone.0297839)
Supplement: S3 Appendix — (DOCX) [file pone.0297839.s003.docx]

**Appendix S3: Details on data processing during analyses**

**Continuous outcome**

In this review, continuous outcomes that were analysed were symptom scores, QOL scores, activity impairment scores, clinical signs, activity impairments, medication scores, and effectiveness/satisfaction scores.

Pooled outcome estimates for continuous data were reported as mean difference (MD) with 95% confidence intervals (CI) if all data were measured using the same measurement scale. Otherwise, standardised mean difference (SMD) and its corresponding CI was used. For MD, individual studies reporting post-treatment mean values and changes in mean from baseline post-treatment were pooled into the same meta-analysis without conversion of data. For SMDs, studies reporting post-treatment means and SD were pooled into separate meta-analyses from studies that reported changes in mean from baseline and SD of the changes. Reasonable estimates of the SD for changes in mean were not calculated as it was agreed among team members that an appropriate correlation coefficient could not be generated from currently available data. Overall, the inverse variance random effects model was applied due to expected high heterogeneity of herbal trials. SDs were estimated from SEs, p values, and 95% CI values wherever necessary and appropriate. In cases where the SD was reported as zero, clarification from authors were sought, and if necessary, an SD of 0.00001 (the smallest number accepted by RevMan5) was used instead.

**Dichotomous outcome**

In this review, dichotomous outcomes that were analysed were responder/improvement rates and the number of patients who needed rescue medications.

Pooled outcome estimates for dichotomous outcomes were generated using the Mantel-Haenszel methods and reported as risk ratios (RRs). Studies with no events in either or both arms were excluded from the analysis as recommended in Section 10.4.4.2 of the Cochrane Handbook for Systematic Reviews of Intervention.

**Deviation from protocol**

Originally, it was planned for pooled analysis to be conducted for the same medicinal plant with more than three studies reporting the same outcome. However, based on the available literature, this approach was not possible and hence pooled analysis was conducted based on comparator and outcomes only. Wherever feasible, subgroup analysis by plant type was carried out.
