# Supplementary material for: Medicinal plants for allergic rhinitis: A systematic review and meta-analysis
Source: PLoS One. 2024 Apr 11;19(4):e0297839. doi: 10.1371/journal.pone.0297839 (PMC11008904; doi:10.1371/journal.pone.0297839)
Supplement: S4 Appendix — (PDF) [file pone.0297839.s004.pdf]

Appendix S4. List of excluded studies after full text screening

Study No	Authors	Year	Title	Reason for exclusion
31	Ansari et al.	2010	Montelukast versus nigella sativa for management of seasonal allergic rhinitis: a single blind comparative clinical trial	Non-randomised
34	Ariaee et al.	2018	Oral Administration of Zataria multiflora Extract Decreases IL-17 Expression in Perennial Allergic Rhinitis	Duplicate study of 35
97	Bhaskaran et al.	2012	Clinical evaluation of Amragandha haridra (Curcuma amada Roxb) in Pratisyaya W.S.R to allergic Rhinitis: a folklore claim	Non-randomised
695	Nikakhlagh et al.	2010	Herbal treatment of allergic rhinitis: the use of Nigella sativa	Non-randomised
804	Ross, S. M.	2016	Allergic Rhinitis: A Proprietary Extract of Pinus pinaster Aiton (Pycnogenol) Is Found to Improve the Symptoms Associated With Allergic Rhinitis	Commentary of 985
835	Schapowal and Merimond	2003	Butterbur extract Ze 339 is effective and safe in treating all symptoms of seasonal allergic rhinitis	Duplicate study of 832
842	Segawa et al.	2007	Clinical effects of a hop water extract on Japanese cedar pollinosis during the pollen season: a double-blind, placebo-controlled trial	Non-randomised
902	Tanticharoenwiwat et al.	2017	Inhibitory effect of Phlai capsules on skin test responses among allergic rhinitis patients: a randomized, three-way crossover study	Intervention duration one day only and no clinical symptoms related outcome
1022	Yazdi et al.	2020	Efficacy of Sweet Violet (Viola odorata) flower oil on the symptoms of adults with allergic rhinitis: A double-blind randomized placebo-controlled clinical trial	Mixture (vehicle used is almond oil, which has its own potentially bioactive phytoconsituents)
982	Wiesenauer and Gaus	1985	Double-blind trial comparing the effectiveness of the homeopathic preparation Galphimia potentiation D6, Galphimia dilution 10(-6) and placebo on pollinosis	Homeopathy
983	Wiesenauer et al.	1983	[Pollinosis therapy with Galphimia glauca]	Homeopathy
1020	Yang et al.	2010	Traditional Chinese medicine, Xin-yi-san, reduces nasal symptoms of patients with perennial allergic rhinitis by its diverse immunomodulatory effects	Mixture
1044	Zhang et al.	2004	[Effect of kebimin decoction in treating allergic rhinitis and on blood levels of nitric oxide and superoxide dismutase]	Mixture
1060	Zhu et al.	2008	Qufeng Decoction for treating allergic rhinitis: a randomized controlled trial	Mixture
1083	Chang et al.	2022	A Houttuynia cordata-based Chinese herbal formula improved symptoms of allergic rhinitis during the COVID-19 pandemic	Mixture
1116	Kim et a.l	2019	A multicenter study on the efficacy and safety of So-Cheong-Ryong-Tang for perennial allergic rhinitis	Mixture
