# Supplementary material for: Medicinal plants for allergic rhinitis: A systematic review and meta-analysis
Source: PLoS One. 2024 Apr 11;19(4):e0297839. doi: 10.1371/journal.pone.0297839 (PMC11008904; doi:10.1371/journal.pone.0297839)
Supplement: S5 Appendix — (DOCX) [file pone.0297839.s005.docx]

**Appendix S5: Characteristics of included study.**

**Table S1. Population characteristics.**

| **Author, Year** | **Study design** | **Sample size (n)** | **Age (Years)^1^** | **% Male** | **AR Subtype** | **AR Severity/Persistence** | **Diagnostic criteria** |
| --- | --- | --- | --- | --- | --- | --- | --- |
| Achararit, 2023 | Cross-over | 25 | 25.96 ± 7.72 | 30.0 | NR | Mild to moderate persistent | Clinical symptoms (TNSS) + skin prick test |
| Atar, 2022 | Parallel | 59 | Intervention: 36 ± 13  Control: 34 ± 7 | 50.8 | NR | Mild persistent | ARIA |
| Ariaee, 2021 | Parallel | 30 | 33 (Range 11–67) | 43.3 | Seasonal | NR | GLORIA |
| Jung, 2021 | Parallel | 60 | Intervention^2^: 33.7 ± 9.6  Control (FEX) ^2^: 35.6 ± 13.7  Control (Placebo) ^2^: 35.5 ± 10.5 | 30.0 | NR | All severity of intermittent and persistent  (Majority mild persistent) | ARIA + skin prick test |
| Yusin, 2021 | Parallel | 45 | Intervention: 48.8 ± 15.9  Intervention + Intranasal corticosteroids: 45.1 ± 12.2  Control 1(Intranasal corticosteroids): 58.6 ± 13.5 Control 2(Placebo): 44.5 ± 18.5 | 75.6 | Seasonal | NR | Clinical symptoms & history + skin prick test |
| Yamprasert, 2020 | Parallel | 72 | Intervention: 35.42 ± 12.73 Control: 30.75 ± 9.72 | 27.5 | NR | Moderate | Clinical symptoms (TNSS) & history |
| Derakhshan. 2019 | Parallel | 77 | Intervention: 33.5±10.26 Control: 34±11.59 | 49.4 | Seasonal | Intermittent | ARIA |
| Steels, 2019 | Parallel | 60 | Overall: 43.25 ± 14.51 Intervention: 42.8 ± 14.82  Control: 43.7 ± 14.19 years | 38.3 | Seasonal | NR | Clinical practice guideline: allergic rhinitis executive summary [1] |
| Arpornchayanon, 2019 | Parallel | 16 | Intervention: 42.88±14.25 Control: 27.00 ± 7.54 | 31.3 | NR | Mixed (mild to severe intermittent or persistent) | Clinical symptoms & history + skin prick test |
| Rezaeian, 2018 | Parallel | 65 | Intervention: 44.12 ± 13.03 Control: 45.50 ± 12.57 | 43.1 | NR | Mild - moderate | Clinical signs, symptoms & history |
| Hajiheydari, 2017 | Parallel | 71 | Intervention: 33.53 ± 12.04 Control: 31.85 ± 13.21 | 33.8 | Perennial | Intermittent | ARIA |
| Bakhshaee. 2017 | Parallel | 40 | Intervention: 23.98 ±10.72  Control: 28.40 ±10.46 | 32.5 | NR | Persistent | NR |
| Walanj, 2014 | Parallel | 38 | Intervention: 32.10 ± 8.93  Control: 39.85 ± 10.81 | 40 | Seasonal | Severe | Clinical symptoms & history (RQLQ score) |
| Yonekura, 2011 | Parallel | 89 | Intervention: 30.5 ± 10.4 Control: 29.9 ± 7.9 | 52.8 | Perennial | Moderate - severe | Clinical symptoms & history |
| Jung, 2011 | Parallel | 59 | Overall: 26.6  Intervention: 25.77 ± 6.20 Control: 27.07 ± 6.84 | 62.8 | Perennial | Persistent | Clinical symptoms & history + skin prick test |
| Matkovic, 2010 | Parallel | 48 | Intervention: 33.3 ± 12.7  Control: 31.5 ± 9.6 | 54.2 | Seasonal | Moderate - severe | Clinical symptoms & history + skin prick test |
| Wilson, 2010 | Parallel | Phase 1: NR Phase 2: 39 | Phase 1: Intervention: 42.0 ± 9.2 Control 43.5 ± 12.8   Phase 2 Intervention: 46.2 ± 12.7  Control: 43.5 ± 13.9 | 35 | Seasonal | NR | Clinical symptoms (TNSS) history + skin prick test |
| Wu, 2009 | Parallel | 191 | Intervention: 5.5 ± 0.22 Control: 5.89 ± 0.17 | 60.2 | NR | Mild - severe | 1. [Guidelines for clinical research of new Chinese medicines: Diagnostic criteria of allergic rhinitis] [2] 2. Skoner DP. Allergic rhinitis: definition, epidemiology, pathophysiology, detection, and diagnosis [3] |
| Cingi, 2008 | Parallel | 129 | Intervention: 30.1 ± 6.69  Control: 29.9 ± 7.66 | 41.9 | NR | NR | Clinical symptoms & history |
| Yoshimura, 2007 | Parallel | 33 | Intervention: 34.7 ± 10.72  Control: 35.4 ± 12.4 | 42.4 | Perennial | Mild - severe | Guidelines for the Management of Allergic Rhinitis in Japan 2002 [4] |
| Schapowal, 2005 | Parallel | 330 | Intervention: 40.7 ± 14.6 Control (FEX): 38.6 ± 14.4 Control (Placebo): 35.0 ± 12.2 | 40.3 | Seasonal | Intermittent | Clinical symptoms & history + skin prick test + RAST |
| Badar, 2005 | Parallel | 71 | Range: 20-60 | 36 | NR | NR | NR |
| Schapowal, 2004 | Parallel | 186 | Intervention (HD): 41 ± 13  Intervention (LD): 43 ± 14  Control: 44 ± 14 | 36 | NR | Intermittent | Clinical symptoms & history + skin prick test + RAST |
| Gray, 2004 | Cross-over | 35 | Intervention:40 ± 41.16  Control:42 ± 13.75 | 40 | NR | Intermittent | Clinical symptoms & history |
| Takano, 2004 | Parallel | 30 | Intervention (HD): 33.1 ± 6.3 Intervention (LD): 32.2 ± 6.6 Placebo: 33 ± 9.3 | 55.2 | Seasonal | Mild | Clinical symptoms & history + positive serum IgE towards allergen |
| Lee, 2004 | Cross-over | 16 | 43±12 | 43.75 | Perennial | Mild | Clinical signs & history + skin prick test |
| Schapowal, 2002 | Parallel | 125 | Intervention: 39 ± 12  Control: 35 ± 14 | 32.8 | Seasonal | NR | Clinical symptoms & history |
| Bernstein, 2002 | Parallel | 51 | Intervention: 43 Control:36 | 37.0 | Seasonal | Moderate - severe | Clinical symptoms & history + skin prick test |
| Mittman, 1990 | Parallel | 69 | Intervention: 34.7 Control: 36.4 | 50.7 | Seasonal | Mild - severe | Clinical symptoms & history |

^1^in mean ± SD unless otherwise stated

^2^data was presented as mean ± SE in original paper, however the calculations for SD based on the formula SD=SE X SQRT(n) did not return reasonable numbers, therefore, data is presented as original paper. The authors did not respond to email query.

Allergic Rhinitis (AR); Allergic Rhinitis and its Impact on Asthma (ARIA); Fexofenadine (FEX) Global Resources in Allergy (GLORIA); Not reported (NR); Rhinoconjunctivitis Quality of Life Questionnaire (RQLQ); Radioallergosorbent test (RAST); Standard deviation (SD); TNSS (Total Nasal Symptom Score)

**Table S2. Intervention and comparator details.**

| **Author** | **Plant name**^1^ | **Plant part** | **Form** | **Route** | **Dose** | **Duration (Days)** | **Add on**^3^ | **Control** | **Dose** | **Rescue medication** |
| --- | --- | --- | --- | --- | --- | --- | --- | --- | --- | --- |
| Achararit, 2023 | *Zingiber cassumunar* Roxb | NR | Standardised extract (Compound D 4 mg/ 100 mg extract) | Oral | 200 mg daily | 28 | No | Placebo | NA | No |
| Atar, 2022 | *Matricaria chamomilla* L. | NR | Liquid extract with isotonic sea water | Intranasal spray | 0.42 mL (3 sprays) each nostril B.I.D | 28 | Yes | Placebo | NA | No |
| Jung, 2021 | Korean red ginseng | NR | Capsule | Oral | 3 mg/kg/day | 28 | No | 1) Fexofenadine 2) Placebo | 1) Fexofenadine -120 mg OD 2) Placebo - NA | No |
| Ariaee, 2021 | *Zataria multiflora* | NR | 20% hydroalcoholic extract syrup (Thymol 20.5 mg/100 mL; carvacrol 2.85 mg/100 mL) | Oral | 10 mL T.I.D | 60 | Yes | Cetrizine tablet and fluticasone nasal spray | NR | No |
| Yusin, 2021 | Brocolli | Seed, sprout | Extract tablet | Oral | 1500 mg Q.D | 21 | Yes^4^ | 1) Intranasal corticosteroids (type NR) 2) Placebo | 1) Intranasal corticosteroids - NR 2) Placebo - NA | No |
| Yamprasert, 2020 | *Zingiber officinale* Roscoe | Rhizome | 95% ethanol extract capsule  (6-gingerol 71.13 mg/g; 6-shogaol 19.65 mg/g) | Oral | 125 mg B.I.D | 42 | No | Loratadine tablet | 10 mg B.I.D | No |
| Derakhshan, 2019 | *Hordeum vulgare* | Seed | Spray-dried standardised decoction powder  (barley: water 1:14; pyrogallol 65 mg/100 g) | Oral | 15 g B.I.D | 14 | No | Fexofenadine | 60 mg B.I.D | No |
| Steels, 2019 | *Cinnamomum zeylanicum* | Bark | Standardised extract nasal spray (Total polyphenols ≥ 40 μg/100 μL) | Intranasal | Two sprays in each nostril B.I.D | 7 | No | Placebo | NA | No |
| Arpornchayanon, 2019 | *Allium ascalonicum* L. | Bulb | Capsule | Oral | 3 g daily | 28 | Yes | Cetrizine dihydrochloride tablet | 10 mg Q.D | No |
| Rezaeian, 2018 | *Nigella sativa* | NR | Nasal spray | Intranasal | 2 puffs (1 g) daily | 56 | Yes | Cetrizine and Azithromycin tablet | Cetrizine 10 mg/day; Azithromycin 500 mg B.I.D x 1/7 then 500mg Q.D | No |
| Hajiheydari, 2017 | *Nepeta bracteata* Benth. (Lamiaceae) | NR | Aqueous extract in syrup (TPC 293 ± 7 mg gallic acid equivalent/100 mL; TFC 258 ± 5 mg rutin equivalent/100 mL) | Oral | 10 mL T.I.D | 28 | No | Placebo | NA | No |
| Bakhshaee, 2017 | *Urtica dioica* L. | Root | Extract tablet | Oral | 15 mg Q.I.D | 30 | Yes | Loratadine | 10 mg T.I.D | No |
| Walanj, 2014 | *Cinnamonum zeylanicum* | Bark | Standardised hydroalcholic extract (Type A proanthocynidin 1 µg/µL) | Intranasal | One spray (100 μg) in each nostril B.I.D | 28 | No | Placebo | NA | No |
| Yonekura, 2011 | *Rubus suavissimus* | NR | Extract capsule (galloyl-oxygen-diphenyl (GOD)-type ellagitannin ~40%) | Oral | 400 mg daily | 28 | No | Placebo | NA | Yes; Fexofenadine |
| Jung, 2011 | Fermented red ginseng | NR | Powder in capsule  (236.7 mg crude saponin, 2.3 mg Rb2, 0.1 mg Rb3, 0.6 mg Rc, 9.5 mg Rd, 0.5 mg Re, 0.6 mg Rg1, 8.2 mg Rg2, 27.7 mg Rg3, 12.1 mg Rh1, 3.1 mg Rh2, and 61.0 mg compound K per gram) | Oral | 750 mg B.I.D | 28 | No | Placebo | NA | No |
| Matkovic, 2010 | *Astragalus membranaceus* (Fisch.) Bunge, Fabaceae | Root | Standardised root extract with herbal and mineral complex  (40% polysaccharides) | Oral | 160 mg B.I.D | 42 | No | Placebo | NA | No |
| Wilson, 2010 | *Pinus pinaster* Ait. | Bark | Standardised bark extract tablet | Oral | 50 mg B.I.D | 21-56 | No | Placebo | NA | Yes; Antihistamines |
| Wu, 2009 | *Flos magnoliae* | NR | Volatile oil nanoliposome nasal drops (5.01 mg in 5 mL) | Intranasal | 1 drop in each nostril B.I.D | 20 | No | Cetrizine tablet | 5 mg Q.D | No |
| Cingi, 2008 | *Spirulina platensis* | Algae | Dried paste tablet | Oral | 2 g Q.D | 147 | No | Placebo | NA | No |
| Yoshimura, 2007 | *Lycopersicon esculentum* Miller | Seed, skin | 60% (v/v) ethyl alcohol extract tablets  (0.2% naringenin chalcone) | Oral | 120 mg T.I.D | 56 | No | Placebo | NA | Yes; Mast cell stabiliser, antihistamine, vasoconstrictor or anti-cholinergic nasal drops, mast cell stabiliser eye drops, topical ocular and nasal steroids |
| Schapowal, 2005 | *Petasites hybridus* | Leaf | Standardised carbon dioxide extract tablet (8mg petasine/tablet) | Oral | One tablet T.I.D^2^ | 14 | No | 1) Fexofenadine tablet 2) Placebo | 1) Fexofenadine - 180 mg Q.D 2) Placebo - NA | No |
| Badar, 2005 | *Tinospora cordifolia* (Willd) Miers | Stem | Standardised aqueous extract tablet | Oral | 300 mg T.I.D | 56 | No | Placebo | NA | No |
| Schapowal, 2004 | *Petasites hybridus* | Leaf | Standardised carbon dioxide extract tablet (8mg petasine/tablet) | Oral | HD: One tablet T.I.D^2^ LD: One tablet B.I.D^2^ | 14 | No | Placebo | NA | No |
| Gray, 2004 | *Petasites hybridus* | NR | Tablet | Oral | 50 mg B.I.D | 14 | No | Placebo | NA | No |
| Takano, 2004 | *Perilla frutescens* | Leaf | 1.0% w/v citric acid extract enriched with rosmarinic acid tablet (20% w/w rosmarinic acid) | Oral | HD: 200mg Q.D LD: 50mg Q.D | 21 | No | Placebo | NA | No |
| Lee, 2004 | *Petasites hybridus* | NR | Capsule | Oral | 50 mg B.I.D | 7 | No | 1) Fexofenadine tablet 2) Placebo | 1) Fexofenadine - 180 mg Q.D 2) Placebo - NA | No |
| Schapowal, 2002 | *Petasites hybridus* | NR | Standardised carbon dioxide extract tablet (8mg petasine/tablet) | Oral | One tablet Q.I.D^2^ | 14 | No | Cetrizine | 10 mg Q.D | No |
| Bernstein, 2002 | Grapeseed | Seed | Standardised extract capsule  (Procyanidolic value >95; total polyphenols 90%) | Oral | 100 mg B.I.D | 56 | No | Placebo | NA | Yes; Chlorpheniramine |
| Mittman, 1990 | *Urtica dioica* | NR | Freeze-dried Urtica dioica in gelatine capsule | Oral | 600 mg (2 doses) at onset of symptoms (Mean number of doses taken/patient= 18.3) | 7 | No | Placebo | NA | No |

^1^Scientific name presented as reported in the source article. If the scientific name is not reported, the common name is presented as reported in the source article

^2^Strength of each tablet not reported

^3^As add-on to conventional therapy

^4^Herbal intervention was investigated both as (1) single intervention and (2) as add-on to conventional therapy

Once daily (Q.D); Twice daily (B.I.D); Three times daily (T.I.D); Four times daily (Q.I.D); High dose (HD); Low dose (LD); Not reported (NR); Not applicable (NA)

**Table S3. Outcome details.**

| **Author** | RQLQ^1^ | SNOT22 | RCAT | NS^2^ | OS | TS | Non-ARS^3^ | SF-36 | WPI | Global assessment^4^ | PNIF | NAR | Lund– McKay Score | Modified Lund Kennedy Score | Clinical Signs^5^ | Sleep quality | Perceived stress | QOL^6^ | Rescue medication | Effectiveness/ Satisfaction score |
| --- | --- | --- | --- | --- | --- | --- | --- | --- | --- | --- | --- | --- | --- | --- | --- | --- | --- | --- | --- | --- |
| Achararit, 2023 |  |  |  | X |  |  |  |  |  |  |  |  |  |  |  |  |  |  |  |  |
| Atar, 2022 |  | X |  |  |  |  |  |  |  |  |  |  |  |  |  |  |  |  |  |  |
| Jung, 2021 |  |  |  | X | X |  | X |  |  |  |  |  |  |  |  |  |  |  |  |  |
| Ariaee, 2021 |  | X |  |  |  |  |  |  |  |  |  |  |  |  |  |  |  |  |  |  |
| Yusin, 2021 |  |  |  | X |  |  |  |  |  |  | X |  |  |  |  |  |  |  |  |  |
| Yamprasert, 2020 | X |  |  | X |  |  |  |  |  |  |  |  |  |  | X |  |  |  |  |  |
| Derakhshan, 2019 |  |  | X | X | X | X | X |  |  | X |  |  |  |  |  |  |  | X |  |  |
| Steels, 2019 | X |  |  | X | X |  | X |  | X |  |  |  |  |  |  | X | X |  |  |  |
| Arpornchayanon, 2019 |  |  |  | X | X |  |  |  |  |  |  | X |  |  |  |  |  |  |  |  |
| Rezaeian, 2018 |  | X |  |  |  |  |  |  |  |  |  |  | X | X |  |  |  |  |  |  |
| Bakhshaee, 2017 |  | X |  |  |  |  |  |  |  |  |  |  |  |  |  |  |  |  |  |  |
| Hajiheydari, 2017 |  |  |  | X | X |  |  |  |  |  |  |  |  |  |  |  |  |  |  |  |
| Walanj, 2014 | X |  |  | X |  |  |  |  | X |  |  |  |  |  |  |  |  |  |  |  |
| Yonekura, 2011 | X |  |  | X |  |  |  |  |  | X |  |  |  |  |  |  |  |  | X |  |
| Jung, 2011 | X |  |  | X |  |  |  |  |  |  |  |  |  |  |  |  |  |  |  |  |
| Matkovic, 2010 | X |  |  | X | X |  |  |  |  | X |  |  |  |  |  |  |  |  |  |  |
| Wilson, 2010 |  |  |  | X | X |  |  |  |  |  |  |  |  |  |  |  |  |  | X |  |
| Wu, 2009 |  |  |  | X |  |  |  |  |  | X |  |  |  |  | X |  |  |  |  |  |
| Cingi, 2008 |  |  |  | X |  |  |  |  |  |  |  |  |  |  |  |  |  |  |  | X |
| Yoshimura, 2007 |  |  |  | X |  |  |  |  |  |  |  |  |  |  | X |  |  | X | X |  |
| Schapowal, 2005 |  |  |  | X | X | X |  |  |  | X |  |  |  |  |  |  |  |  |  |  |
| Badar, 2005 |  |  |  | X |  |  |  |  |  | X |  |  |  |  |  |  |  |  |  |  |
| Schapowal, 2004 |  |  |  | X | X |  |  |  |  | X |  |  |  |  |  |  |  |  |  |  |
| Gray, 2004 | X |  |  | X | X |  |  |  |  |  | X |  |  |  |  |  |  |  |  |  |
| Takano,2004 |  |  |  | X | X |  |  |  |  | X |  |  |  |  |  |  |  |  |  |  |
| Lee, 2004 |  |  |  | X |  |  |  |  |  |  | X |  |  |  |  |  |  |  |  |  |
| Schapowal, 2002 |  |  |  |  |  |  |  | X |  | X |  |  |  |  |  |  |  |  |  |  |
| Bernstein, 2002 | X |  |  | X | X |  |  |  |  | X |  |  |  |  |  |  |  |  | X |  |
| Mittman, 1990 |  |  |  |  |  |  |  |  |  | X |  |  |  |  |  |  |  |  |  | X |

^1^RQLQ includes RQLQ, mini RQLQ, JRQLQ

^2^Includes symptoms scores using TNSS, VAS; and symptom duration

^3^Non ARS refers to non-allergic rhinitis specific symptoms such as headache, mental function

^4^Includes global assessment scores on improvement in overall symptoms and/or percentage of subjects with overall improvement

^5^Includes clinical examination and/or measurements of nasal cavity

^6^Includes QOL measurements that are not measured by RQLQ and SF-36

Rhinoconjunctivitis Quality of Life Questionnaire (RQLQ); Japanese Rhinoconjunctivitis Quality of Life Questionnaire (JRQLQ)Sino-nasal Outcome Test (SNOT22); Rhinitis Control Assessment Test (RCAT); Nasal symptom (NS); Ophthalmology symptom (OS); Throat symptom (TS); Non-allergic rhinitis symptom (Non-ARS); 36-Item Short Form Survey (SF-36), Peak Nasal Inspiratory Flow (PNIF), Nasal Airway Resistance (NAR), Quality of life (QOL)

**References:**

1. Seidman MD, Gurgel RK, Lin SY, Schwartz SR, Baroody FM, Bonner JR, et al. Clinical practice guideline: Allergic rhinitis. Otolaryngol Head Neck Surg. 2015;152(1_suppl):S1-S43. doi:<https://doi.org/10.1177/0194599814561600>.

2. Zhen X. Guidelines for clinical research of new Chinese medicine. China Medical Science and Technology Publishing House, Beijing, China. 2002:162-82.

3. Skoner DP. Allergic rhinitis: Definition, epidemiology, pathophysiology, detection, and diagnosis. J Allergy Clin Immunol. 2001;108(1 Suppl):S2-8. doi:<https://doi.org/10.1067/mai.2001.115569>.

4. Okubo K, Kurono Y, Ichimura K, Enomoto T, Okamoto Y, Kawauchi H, et al. Japanese guidelines for allergic rhinitis 2020. Allergol Int. 2020;69(3):331-45. doi:<https://doi.org/10.1016/j.alit.2020.04.001>.
