# Supplementary material for: Medicinal plants for allergic rhinitis: A systematic review and meta-analysis
Source: PLoS One. 2024 Apr 11;19(4):e0297839. doi: 10.1371/journal.pone.0297839 (PMC11008904; doi:10.1371/journal.pone.0297839)
Supplement: S6 Appendix — (DOCX) [file pone.0297839.s006.docx]

**Appendix S6: Unpublished clinical trials**

| **No.** | **Title of Study** | **Registered Year** | **Registry Number** | **Last Update Date on the Trial Registry** | **Status** |
| --- | --- | --- | --- | --- | --- |
| 1 | A randomized, double-blind, placebo-controlled, cross-over phase IIa/b study to assess the efficacy and safety of 2 dosages of a herbal medicinal product (dry extract BNO-1355) in patients with seasonal allergic rhinitis - BNO-1355 Allergic Rhinitis | 2010 | 2010-018786-33 | Not available | Completed, no published results |
| 2 | Efficacy and safety of Phlai capsule compared to placebo as the treatment in allergic rhinitis patients | 2019 | NCT04182919 | 28/1/2022 | Completed, planned for publication  Date completed: 20/12/2021 |
| 3 | Efficacy of Phlai capsule and loratadine as treatment in allergic rhinitis patients | 2017 | TCTR20171111002 | 11/11/2017 | Completed, no published results   Date completed: 28/02/2018 |
| 4 | Evaluation of the effects of solution made with flax seed oil in the treatment of rhinosinusitis | 2021 | IRCT20210421051030N1 | 30/8/2021 | Ongoing |
| 5 | Efficacy and Safety of Bojungikgi-tang for Persistent Allergic Rhinitis: Study Protocol for a Randomized, Double-blind, Placebo-controlled, Phase II Trial | 2021 | KCT0006616 | 1/11/2021 | Ongoing, recruiting |
| 6 | The Effects of Using Yupingfeng Powder With Variation for the Treatment of Allergic Rhinitis | 2021 | NCT04976023 | 27/06/2022 | Ongoing, recruiting |
| 7 | Randomized controlled clinical study of ''allergy Kang'' regulating body in treatment of allergic rhinitis | 2023 | ChiCTR2300068155 | 23/06/2023 | Not yet recruiting |
| 8 | A study to evaluate the role of Palandwadi nasal spray in the management of rhinitis. | 2022 | CTRI/2022/06/043619 | 9/6/2022 | Ongoing, recruiting |
| 9 | Evaluation of the effects of topical application of micro emulsion prepared with Flax seed oil carrier of lipophilic active ingredients in the treatment of inflammatory disorders of rhino sinus cavity | 2021 | IRCT20210421051030N1 | 13/08/2021 | Recruitment complete |
| 10 | The effect of nasal spray based on herbal extracts (traditional Persian medicine product) compared to placebo on the symptoms of patients with allergic rhinitis | 2022 | IRCT20220517054885N1 | 2/10/2022 | Not yet recruiting |
