# Supplementary material for: Medicinal plants for allergic rhinitis: A systematic review and meta-analysis
Source: PLoS One. 2024 Apr 11;19(4):e0297839. doi: 10.1371/journal.pone.0297839 (PMC11008904; doi:10.1371/journal.pone.0297839)
Supplement: S7 Appendix — (DOCX) [file pone.0297839.s007.docx]

**Appendix S7. Supplementary figures of Forest plots.**

(a) Medicinal plant vs placebo


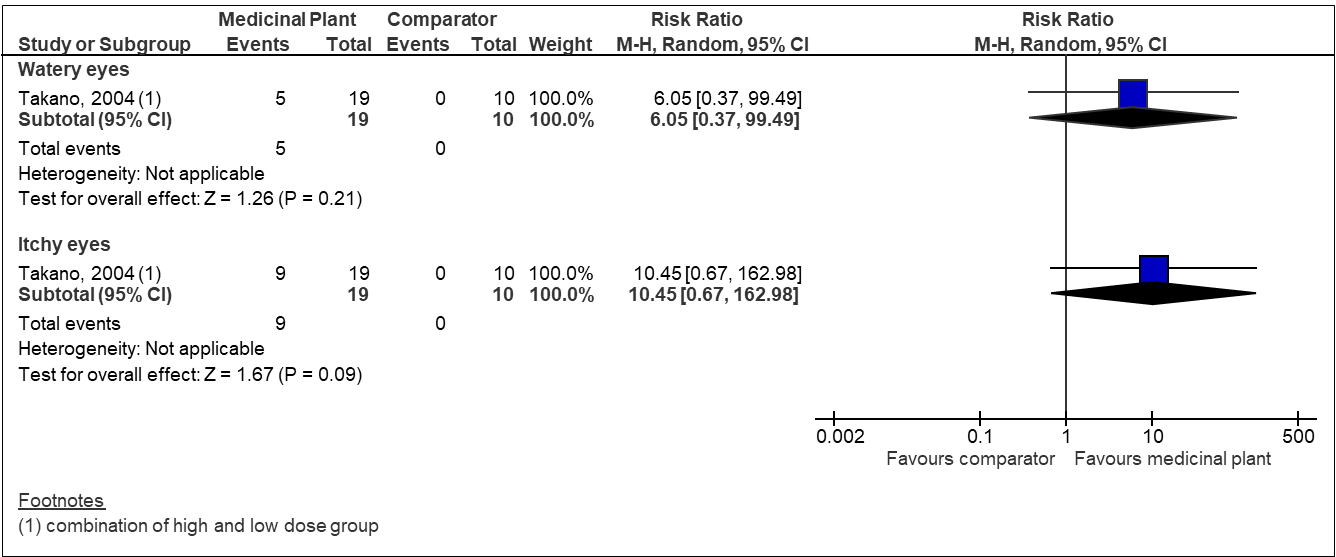


Fig S1. Forest plot of comparison: Medicinal plant vs negative control (placebo), outcome: Responder rates with improvements in individual eye symptoms.


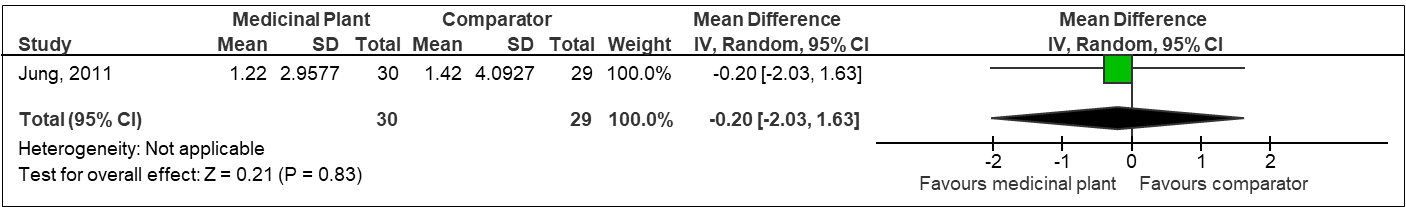


Fig S2. Forest plot of comparison: Medicinal plant vs negative control (placebo), outcome: Symptom duration score.


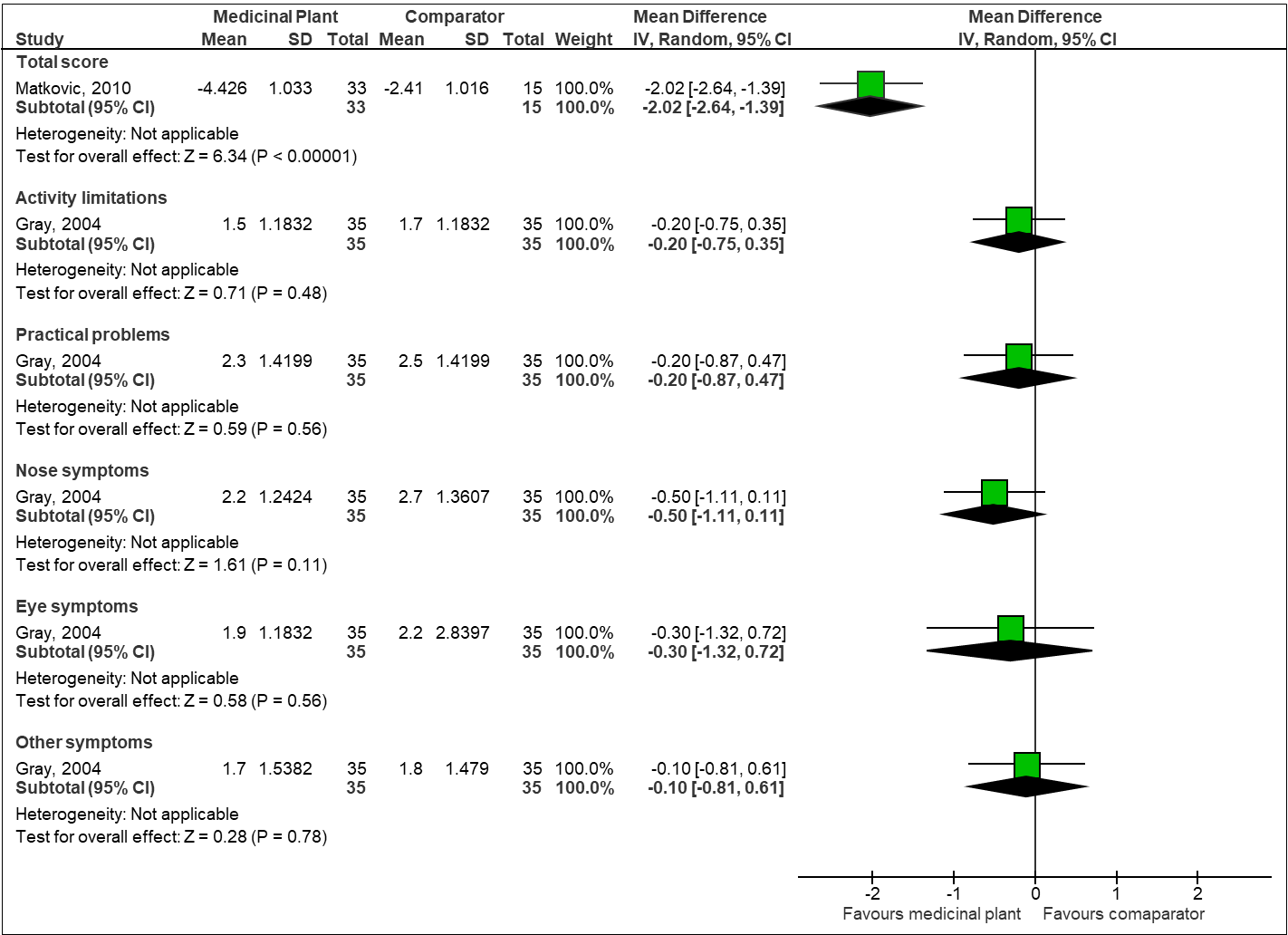


Fig S3. Forest plot of comparison: Medicinal plant vs negative control (placebo), outcome: Mini-RQLQ.


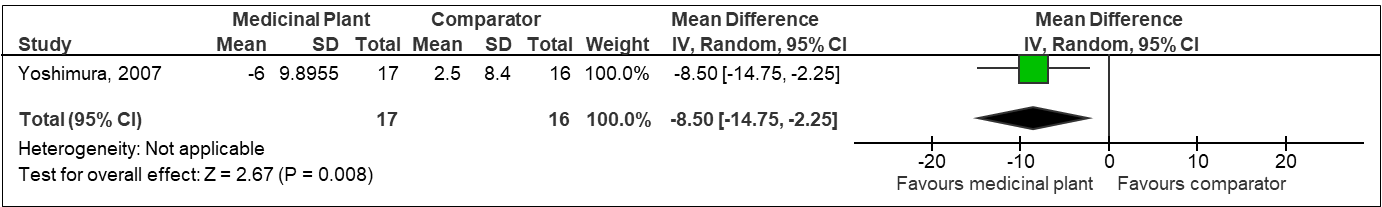


Fig S4. Forest plot of comparison: Medicinal plant vs negative control (placebo), outcome: Other QOL score.


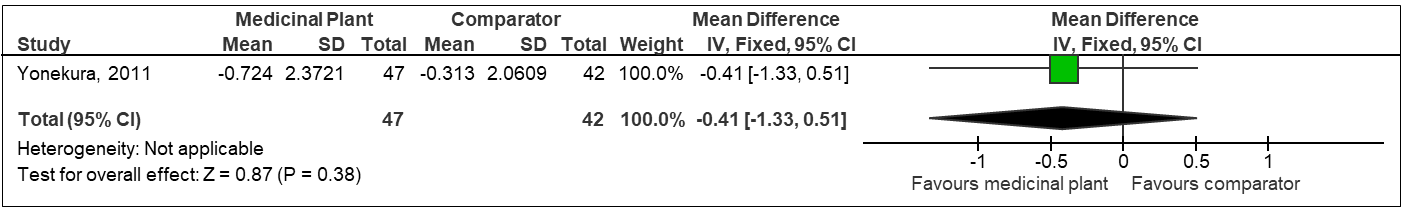


Fig S5. Forest plot of comparison: Medicinal plant vs negative control (placebo), outcome: Medication use score.


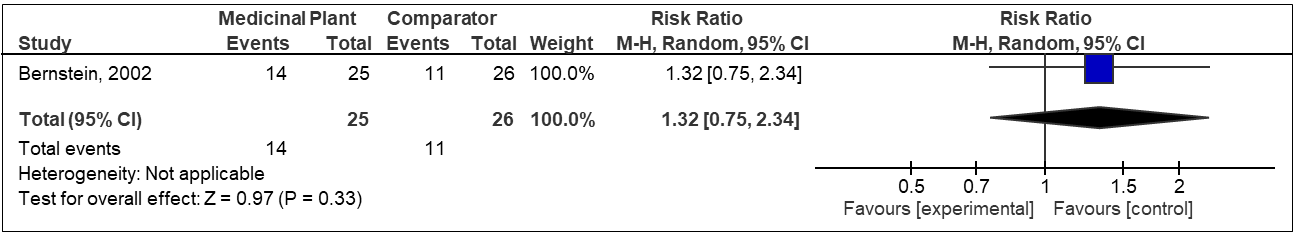


Fig S6. Forest plot of comparison: Medicinal plant vs negative control (placebo), outcome: Patients needing rescue medication.


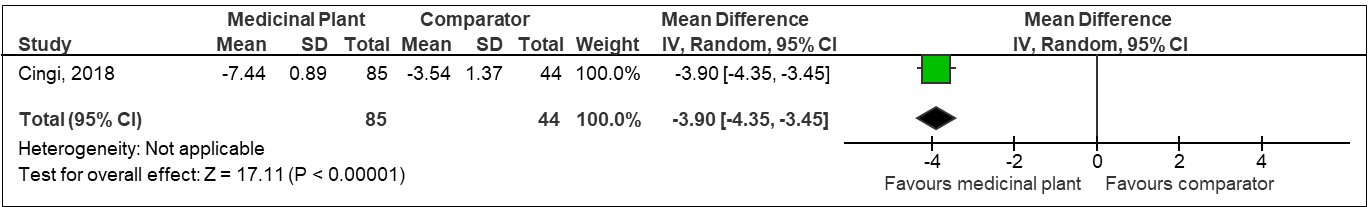


Fig S7. Forest plot of comparison: Medicinal plant vs negative control (placebo), outcome: Effectiveness score.


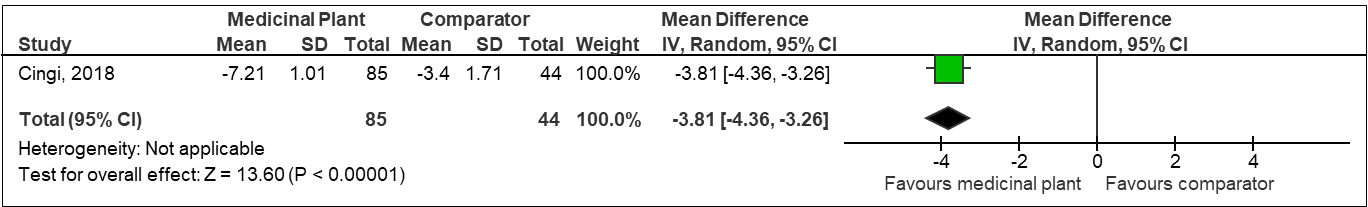


Fig S8. Forest plot of comparison: Medicinal plant vs negative control (placebo), outcome: Satisfaction score.

(b) Medicinal plant vs antihistamine


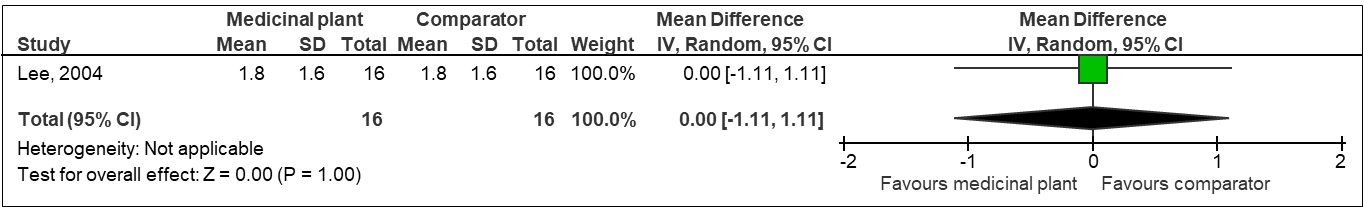


Fig S9. Forest plot of comparison: Medicinal plant vs antihistamine, outcome: Total nasal symptom score (changes in mean).


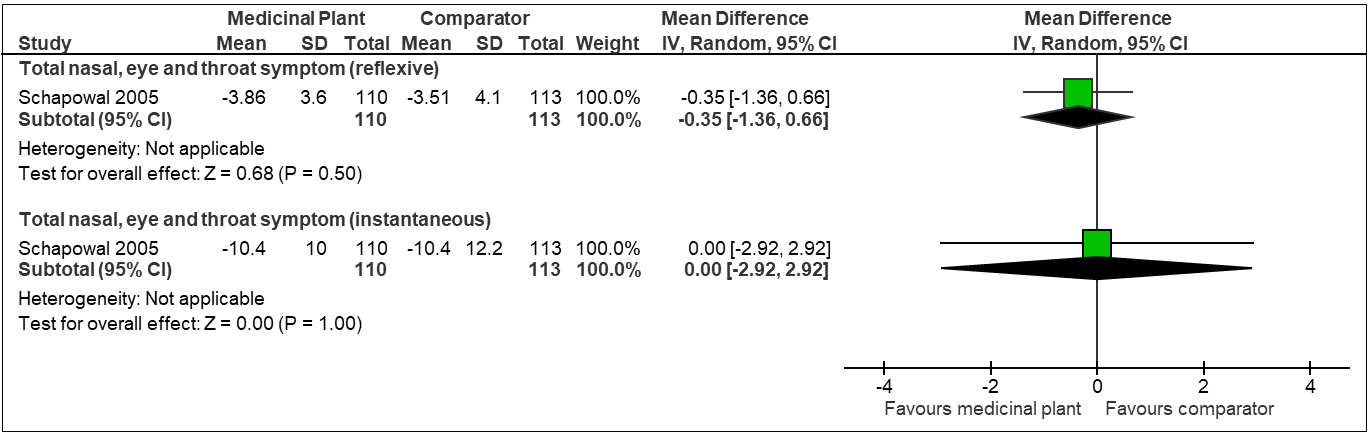


Fig S10. Forest plot of comparison: Medicinal plant vs antihistamine, outcome: Total nasal, eye, and throat symptoms (reflexive and instantaneous).


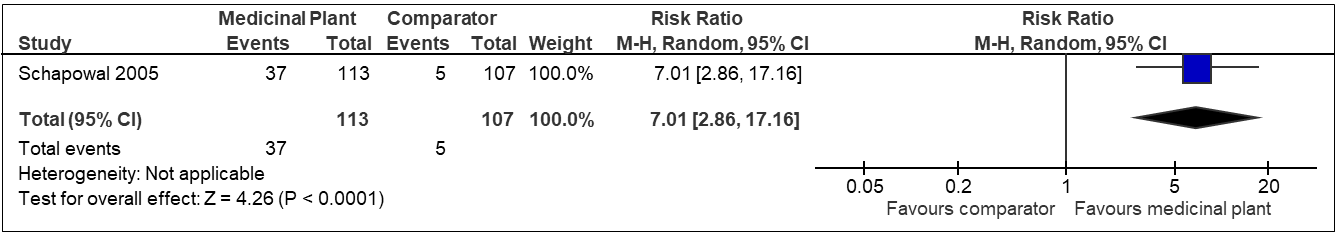


Fig S11. Forest plot of comparison: Medicinal plant vs antihistamine, outcome: Global symptoms assessment (responder rates).


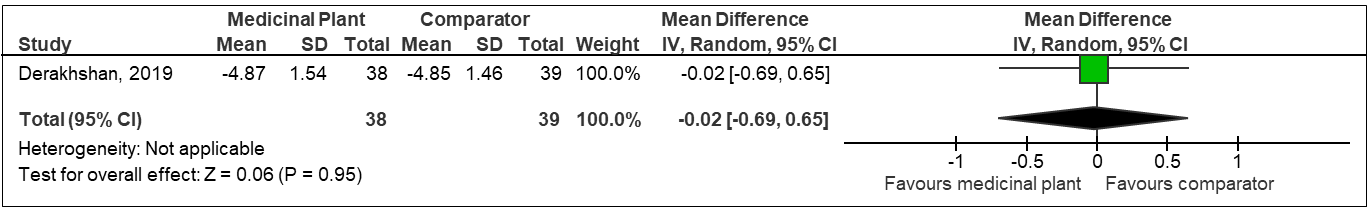


Fig S12. Forest plot of comparison: Medicinal plant vs antihistamine, outcome: Global symptoms assessment score.


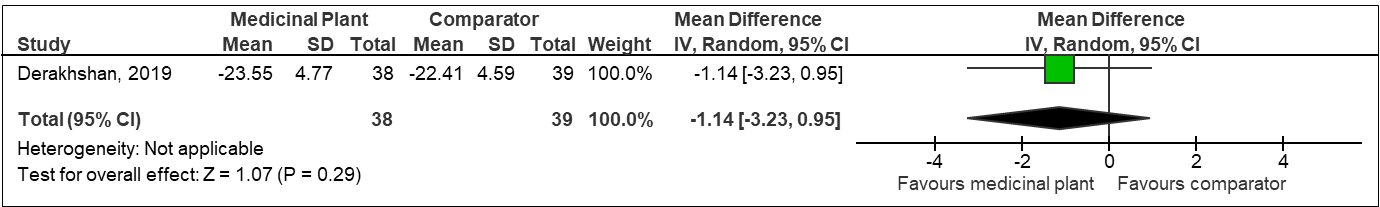


Fig S13. Forest plot of comparison: Medicinal plant vs antihistamine, outcome: RCAT score.


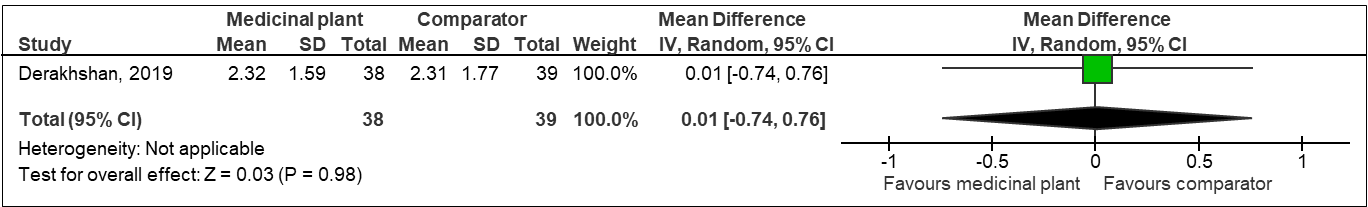


Fig S14. Forest plot of comparison: Medicinal plant vs antihistamine, outcome: Throat symptoms.


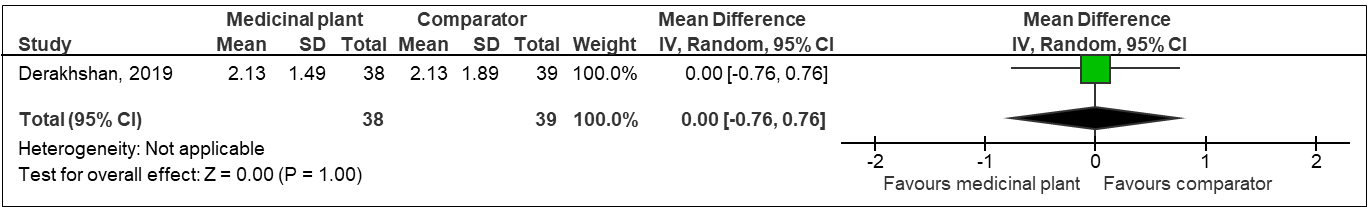


Fig S15. Forest plot of comparison: Medicinal plant vs antihistamine, outcome: Ear symptoms.


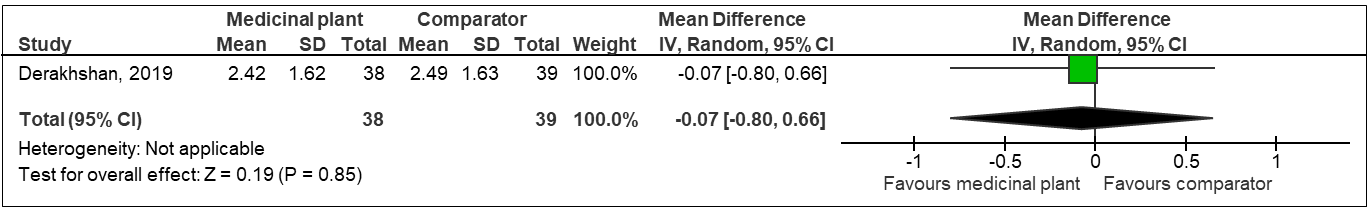


Fig S16. Forest plot of comparison: Medicinal plant vs antihistamine, outcome: Post nasal drip.


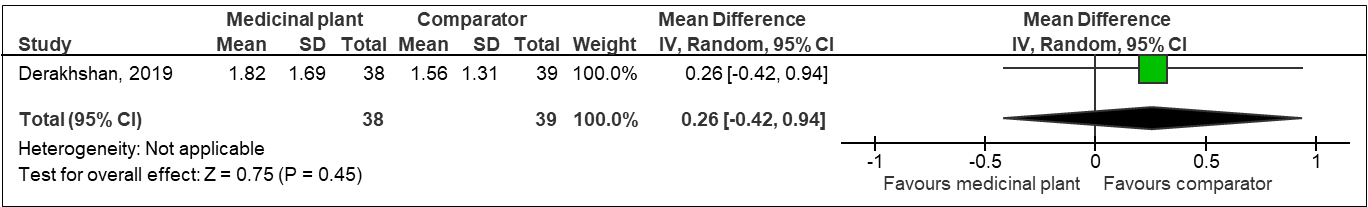


Fig S17. Forest plot of comparison: Medicinal plant vs antihistamine, outcome: Headache.


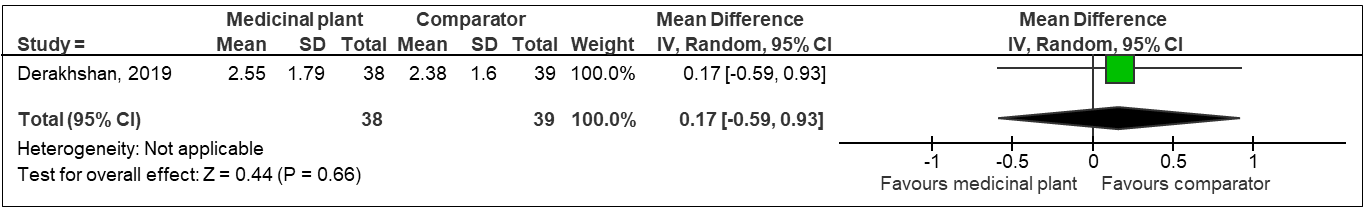


Fig S18. Forest plot of comparison: Medicinal plant vs antihistamine, outcome: Mental function.


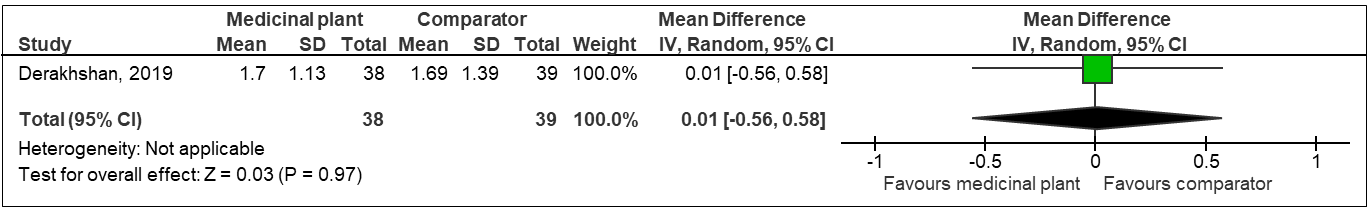


Fig S19. Forest plot of comparison: Medicinal plant vs antihistamine, outcome: Cough.


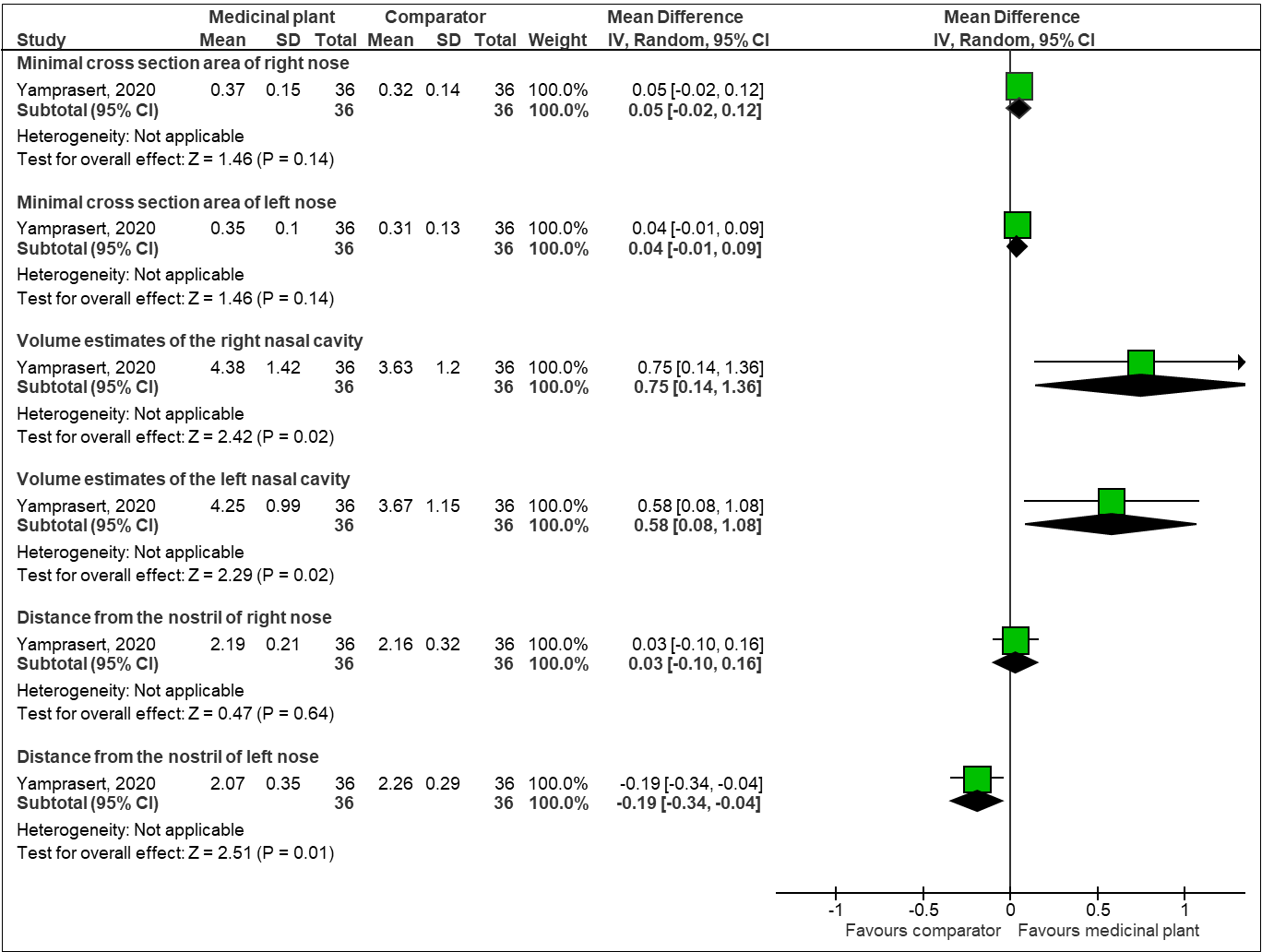


Fig S20. Forest plot of comparison: Medicinal plant vs antihistamine, outcome: Measurement of cross-sectional area of nasal cavities.


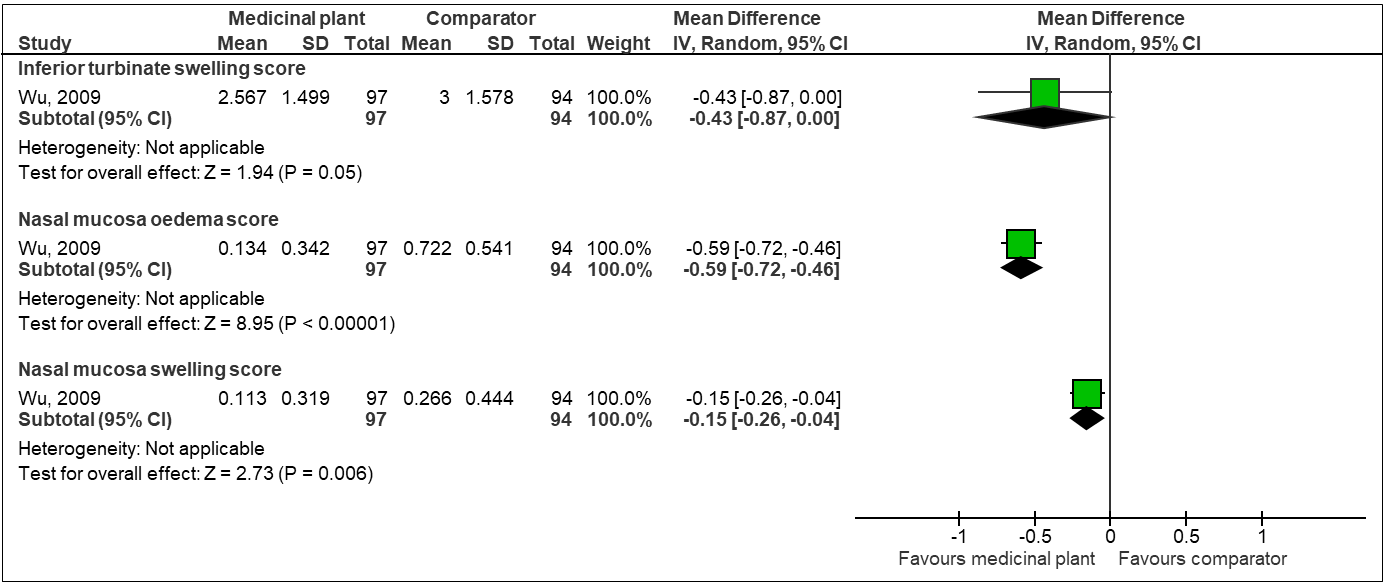


Fig S21. Forest plot of comparison: Medicinal plant vs antihistamine, outcome: Physical appearance of nasal cavities.


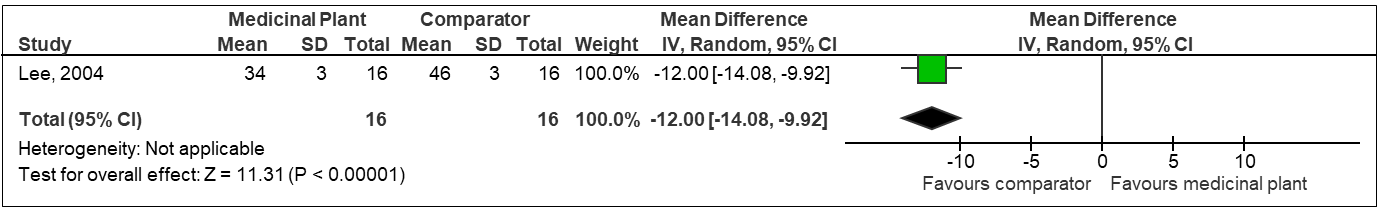


Fig S22. Forest plot of comparison: Medicinal plant vs antihistamine, outcome: PNIF (For study Lee 2004).


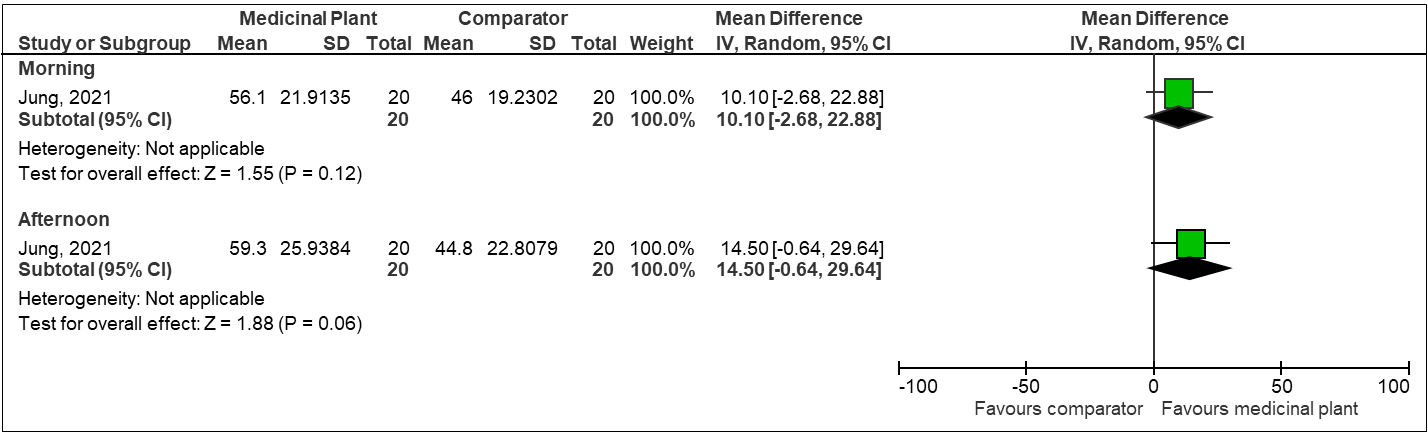


Fig S23. Forest plot of comparison: Medicinal plant vs antihistamine, outcome: PNIF For study Jung 2021).


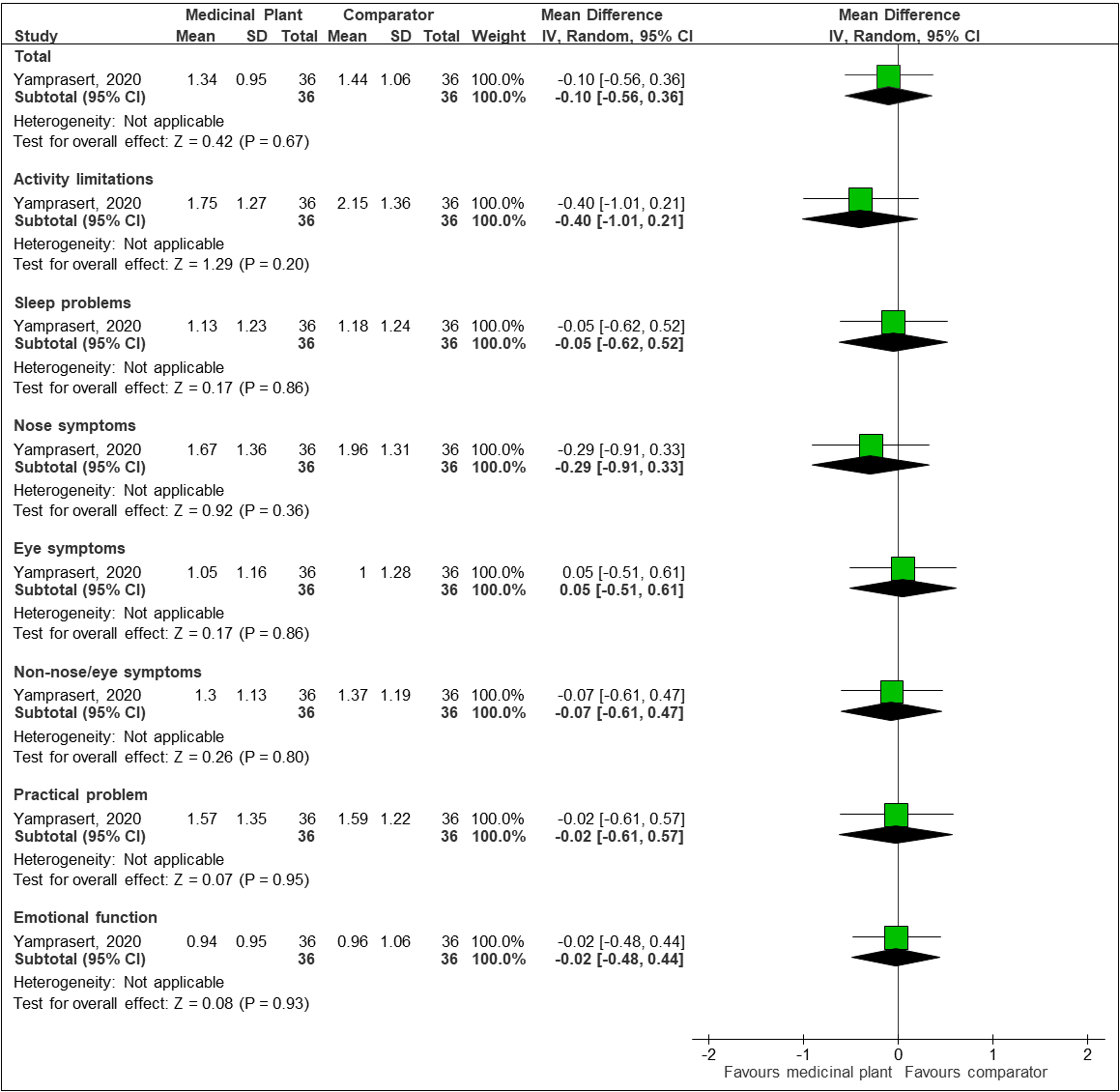


Fig S24. Forest plot of comparison: Medicinal plant vs antihistamine, outcome: RQLQ.


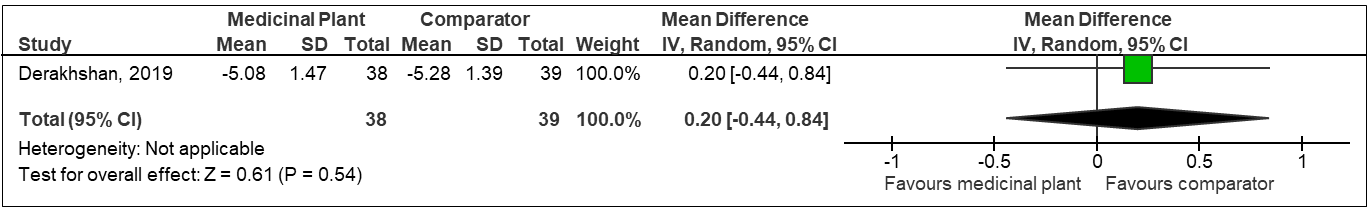


Fig S25. Forest plot of comparison: Medicinal plant vs antihistamine, outcome: Other QOL score (unspecified).

(c) Medicinal plant vs intranasal corticosteroid


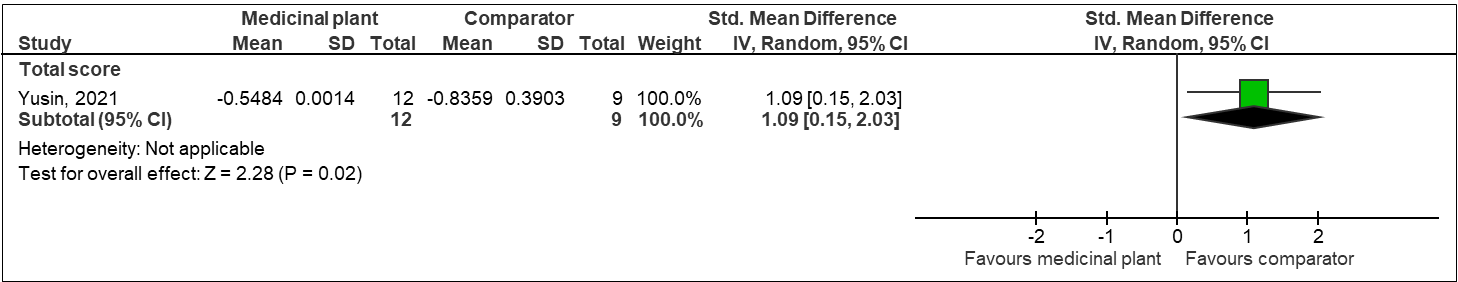


Fig S26. Forest plot of comparison: Medicinal plant vs intranasal corticosteroid, outcome: Total nasal symptom score.

(d) Medicinal plant as add-on to standard treatment vs standard treatment


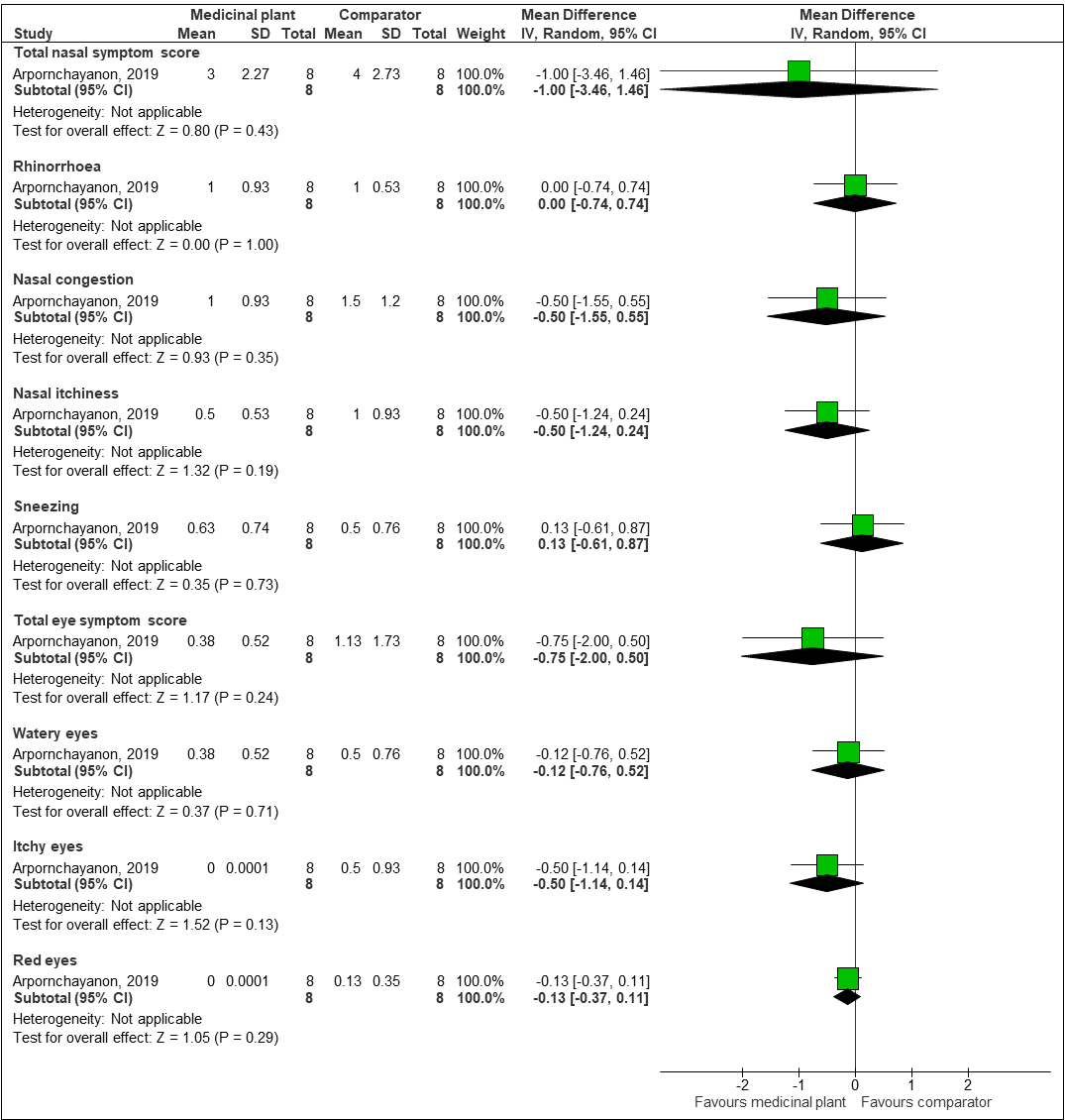


Fig S27. Forest plot of comparison: Medicinal plant as add-on to standard treatment vs standard treatment, outcome: Nasal and eye symptom score (post treatment mean).


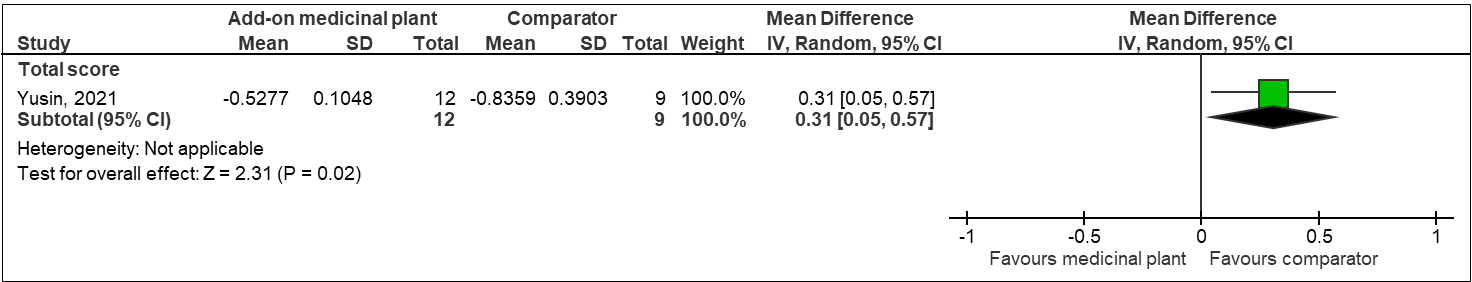


Fig S28. Forest plot of comparison: Medicinal plant as add-on to conventional therapy vs conventional therapy, outcome: Total nasal symptom score (changes in mean).


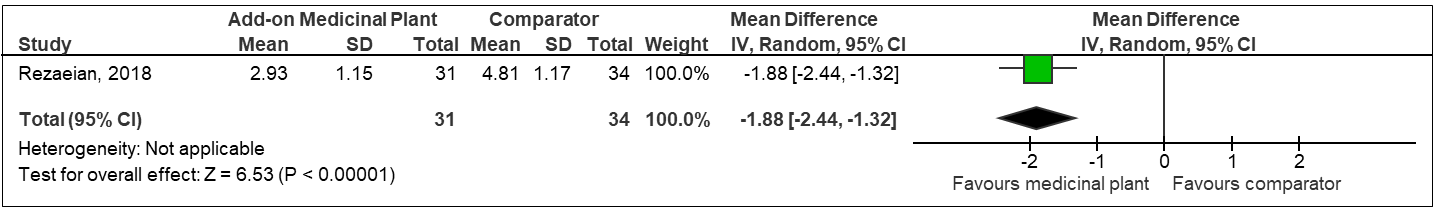


Fig S29. Forest plot of comparison: Medicinal plant as add-on to conventional therapy vs conventional therapy, outcome: Lund-Mckay score.


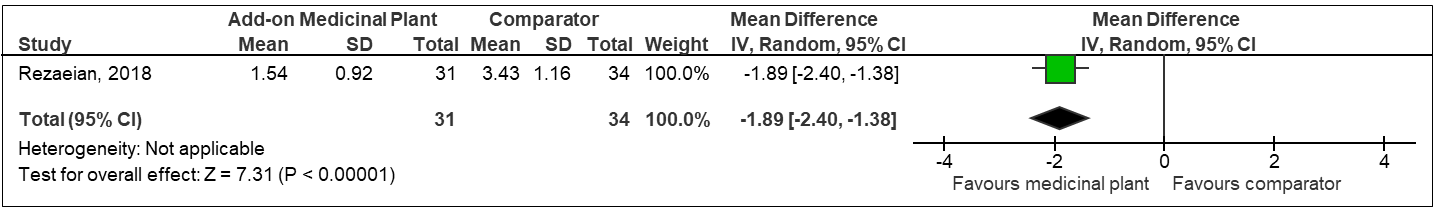


Fig S30. Forest plot of comparison: Medicinal plant as add-on to conventional therapy vs conventional therapy, outcome: Modified Lund Kennedy score.


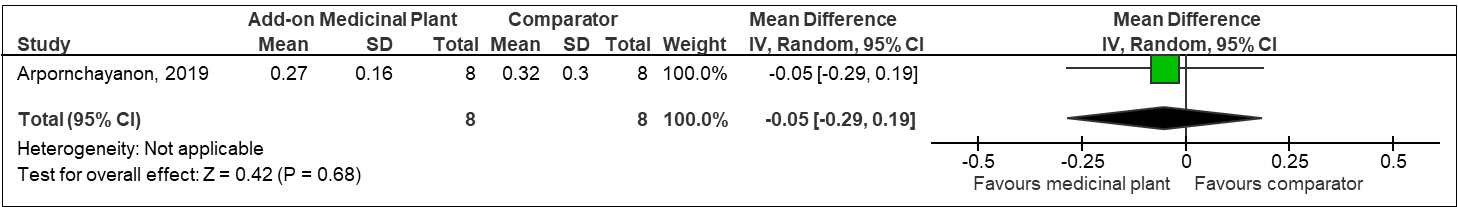


Fig S31. Forest plot of comparison: Medicinal plant as add-on to conventional therapy vs conventional therapy, outcome: Nasal airway resistance.
