# Supplementary material for: Medicinal plants for allergic rhinitis: A systematic review and meta-analysis
Source: PLoS One. 2024 Apr 11;19(4):e0297839. doi: 10.1371/journal.pone.0297839 (PMC11008904; doi:10.1371/journal.pone.0297839)
Supplement: S8 Appendix — (DOCX) [file pone.0297839.s008.docx]

**Appendix S8. Forest plot comparison of medicinal plant vs placebo for RQLQ (without exclusion of Steels, 2019)**

*
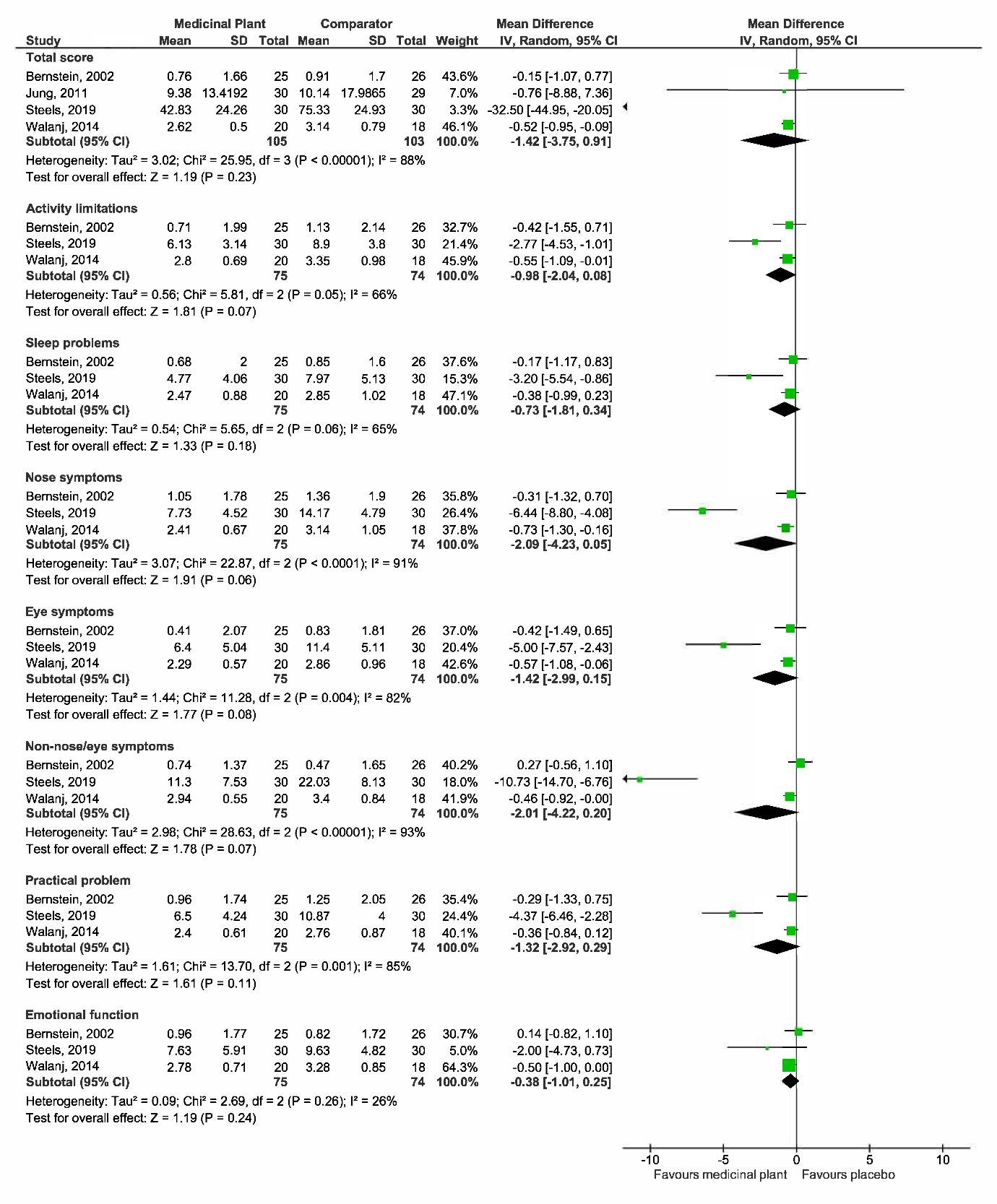
*

**Fig S1. Forest plot comparison of medicinal plant vs placebo for RQLQ (without exclusion of Steels, 2019)**
