# Supplementary material for: Medicinal plants for allergic rhinitis: A systematic review and meta-analysis
Source: PLoS One. 2024 Apr 11;19(4):e0297839. doi: 10.1371/journal.pone.0297839 (PMC11008904; doi:10.1371/journal.pone.0297839)
Supplement: S9 Appendix — (DOCX) [file pone.0297839.s009.docx]

**Appendix S9: Subgroup analysis (Medicinal plant vs oral antihistamines- symptom scores)**


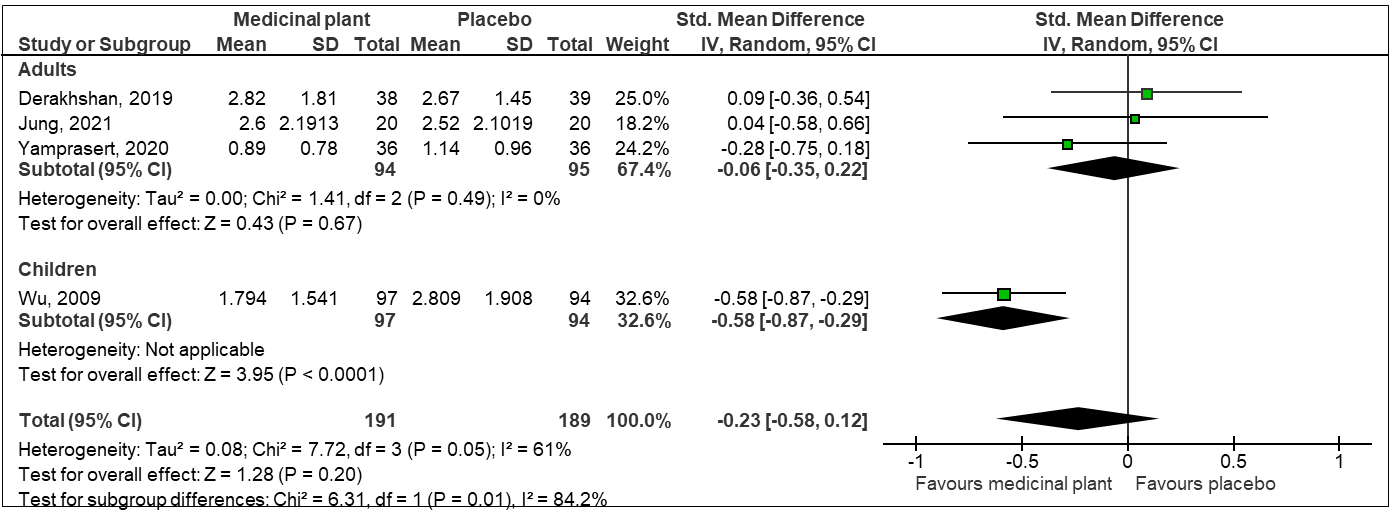


**Fig S1. Subgroup analysis by age/formulation for outcome: Rhinorrhoea.**


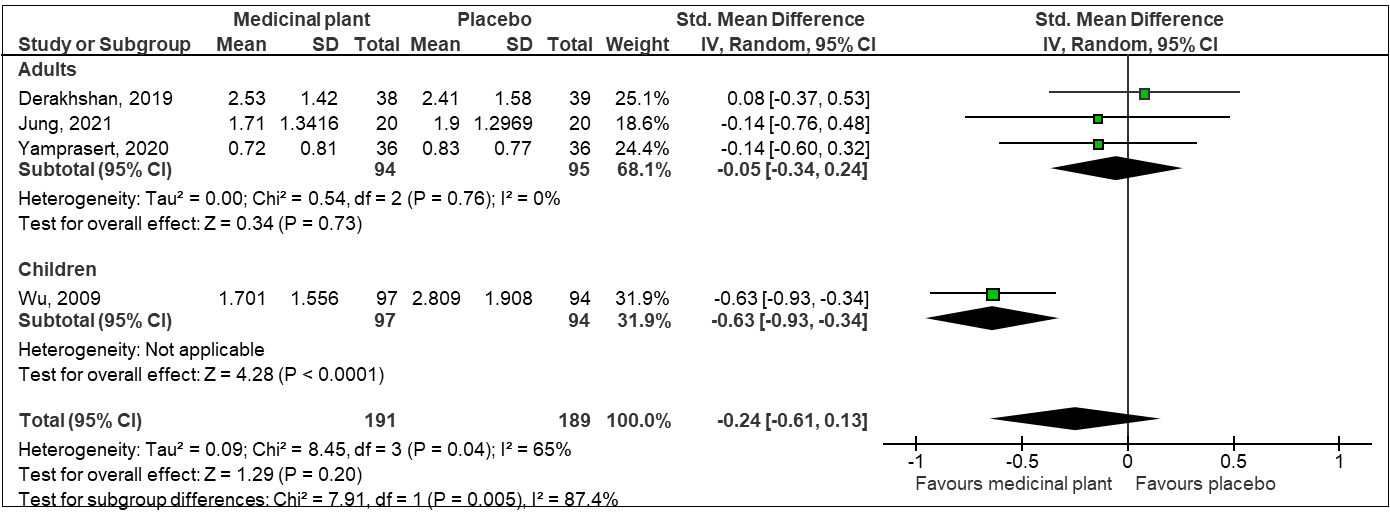


**Fig S2. Subgroup analysis by age/formulation for outcome: Sneezing.**
