# Supplementary material for: Medicinal plants for allergic rhinitis: A systematic review and meta-analysis
Source: PLoS One. 2024 Apr 11;19(4):e0297839. doi: 10.1371/journal.pone.0297839 (PMC11008904; doi:10.1371/journal.pone.0297839)
Supplement: S10 Appendix — (DOCX) [file pone.0297839.s010.docx]

| **Appendix S10. Summary of findings table.**  **Table S1. Medicinal plant compared to negative control (placebo) for Allergic Rhinitis** | | | | | | |
| --- | --- | --- | --- | --- | --- | --- |
| **Medicinal plant compared to negative control (placebo) for Allergic Rhinitis** | | | | | | |
| **Patient or population:** Allergic Rhinitis  **Setting:** Hospital and other specified settings  **Intervention:** Medicinal plant  **Comparison:** Negative control (placebo) | | | | | | |
| Outcomes | **Anticipated absolute effects^*^** (95% CI) | | Relative effect (95% CI) | № of participants (studies) | Certainty of the evidence (GRADE) | Comments |
|  | **Risk with negative control (placebo)** | **Risk with Medicinal plant** |  |  |  |  |
| Nasal and eye symptoms score (post treatment mean) - Total nasal and eye symptoms | - | SMD **0.45 lower** (1.53 lower to 0.63 higher) | - | 111 (2 RCTs) | ⨁◯◯◯ Very low^a,b,c^ | The evidence is very uncertain about the effect of medicinal plant on nasal and eye symptoms score (post treatment mean) - Total nasal and eye symptoms. |
| Nasal and eye symptoms score (post treatment mean) - Total night-time nasal and eye symptoms | The mean nasal and eye symptoms score (post treatment mean) - Total night-time nasal and eye symptoms was **0** | MD **1.37 lower** (2.41 lower to 0.33 lower) | - | 60 (1 RCT) | ⨁⨁⨁◯ Moderate^d^ | Medicinal plant likely results in a slight reduction in nasal and eye symptoms score (post treatment mean) - Total night-time nasal and eye symptoms. |
| Nasal and eye symptoms score (post treatment mean) - Total nasal symptoms | - | SMD **0.31 lower** (0.59 lower to 0.01 lower) | - | 249 (5 RCTs) | ⨁⨁◯◯ Low^a,e^ | Medicinal plant may result in little to no difference in nasal and eye symptoms score (post treatment mean) - Total nasal symptoms. |
| Nasal and eye symptoms score (post treatment mean) - Total day-time nasal symptoms | The mean nasal and eye symptoms score (post treatment mean) - Total day-time nasal symptoms was **0** | MD **5.47 lower** (7.85 lower to 3.09 lower) | - | 60 (1 RCT) | ⨁⨁⨁◯ Moderate^f^ | Medicinal plant likely results in a reduction in nasal and eye symptoms score (post treatment mean) - Total day-time nasal symptoms. |
| Nasal and eye symptoms score (post treatment mean) - Rhinorrhoea | - | SMD 0.85 **lower** (1.73 lower to 0.04 higher) | - | 399 (6 RCTs) | ⨁◯◯◯ Very low^a,g,h^ | Medicinal plant may have no effect on nasal and eye symptoms score (post treatment mean) - Rhinorrhoea but the evidence is very uncertain. |
| Nasal and eye symptoms score (post treatment mean) - Nasal congestion | - | SMD 0.96 **lower** (1.69 lower to 0.23 lower) | - | 585 (7 RCTs) | ⨁◯◯◯ Very low^a,g,i^ | Medicinal plant may reduce nasal and eye symptoms score (post treatment mean) - Nasal congestion but the evidence is very uncertain. |
| Nasal and eye symptoms score (post treatment mean) - Nasal itchiness | - | SMD **0.82 lower** (1.71 lower to 0.07 higher) | - | 399 (6 RCTs) | ⨁◯◯◯ Very low^a,g,j^ | Medicinal plant may have no effect on nasal and eye symptoms score (post treatment mean) - Nasal itchiness but the evidence is very uncertain. |
| Nasal and eye symptoms score (post treatment mean)- sneezing | - | SMD **0.36 lower** (2.49 lower to 0.52 lower) | - | 329 (5 RCT) | ⨁◯◯◯ Very Low^g,k,q^ | Medicinal plant results in little to no difference in nasal and eye symptoms score (post treatment mean)- sneezing but the evidence is very uncertain. |
| Nasal and eye symptoms score (post treatment mean) - Total eye symptoms | - | SMD **0.58 lower** (1.74 lower to 0.57 higher) | - | 141 (2 RCTs) | ⨁◯◯◯ Very low^a,c,m^ | Medicinal plant may have no effect on nasal and eye symptoms score (post treatment mean) - Total eye symptoms but the evidence is very uncertain. |
| Nasal and eye symptoms score (post treatment mean) - Total day-time eye symptoms | The mean nasal and eye symptoms score (post treatment mean) - Total day-time eye symptoms was **0** | MD **2.73 lower** (5.05 lower to 0.41 lower) | - | 60 (1 RCT) | ⨁⨁⨁◯ Moderate^n^ | Medicinal plant likely results in a slight reduction in nasal and eye symptoms score (post treatment mean) - Total day-time eye symptoms. |
| Nasal and eye symptoms score (post treatment mean) - Watery eyes | The mean nasal and eye symptoms score (post treatment mean) - Watery eyes was **0** | MD **0.13 higher** (0.2 lower to 0.46 higher) | - | 51 (1 RCT) | ⨁⨁◯◯ Low^k,o^ | The evidence suggests that medicinal plant does not increase nasal and eye symptoms score (post treatment mean) - Watery eyes. |
| Nasal and eye symptoms score (post treatment mean) - Itchy eyes | The mean nasal and eye symptoms score | MD **0.08 higher** (0.33 lower to 0.49 higher) | - | 91 (2 RCT) | ⨁⨁◯◯ Low^k,o^ | The evidence suggests that medicinal plant does not increase nasal and eye symptoms score (post treatment mean) - Itchy eyes. |
| Nasal and eye symptoms score (post treatment mean) - Red eyes | The mean nasal and eye symptoms score (post treatment mean) - Red eyes was **0** | MD **0.07 higher** (0.23 lower to 0.37 higher) | - | 51 (1 RCT) | ⨁⨁◯◯ Low^k,o^ | The evidence suggests that medicinal plant does not increase nasal and eye symptoms score (post treatment mean) - Red eyes. |
| Nasal, eye, and throat symptoms scores (changes in mean) - Total nasal, eye and throat symptom (Reflexive) | The mean nasal, eye, and throat symptoms scores (changes in mean) - Total nasal, eye and throat symptom (Reflexive) was **0** | MD **3.46 lower** (4.33 lower to 2.59 lower) | - | 217 (1 RCT) | ⨁⨁⨁◯ Moderate^p^ | Medicinal plant likely results in a reduction in nasal, eye, and throat symptoms scores (changes in mean) - Total nasal, eye and throat symptom (Reflexive). |
| Nasal, eye, and throat symptoms scores (changes in mean) - Total nasal, eye and throat symptom (Instantaneous) | The mean nasal, eye, and throat symptoms scores (changes in mean) - Total nasal, eye and throat symptom (Instantaneous) was **0** | MD **8.3 lower** (10.64 lower to 5.96 lower) | - | 217 (1 RCT) | ⨁⨁⨁◯ Moderate^p^ | Medicinal plant probably results in a large reduction in nasal, eye, and throat symptoms scores (changes in mean) - Total nasal, eye and throat symptom (Instantaneous). |
| Nasal, eye, and throat symptoms scores (changes in mean) - Total nasal symptoms | - | SMD **3.09 lower** (5.67 lower to 0.5 lower) | - | 101 (3 RCTs) | ⨁◯◯◯ Very low^a,g,q^ | Medicinal plant may reduce nasal, eye, and throat symptoms scores (changes in mean) - Total nasal symptoms but the evidence is very uncertain. |
| Nasal, eye, and throat symptoms scores (changes in mean) - Rhinorrhoea | - | SMD **0.34 lower** (0.6 lower to 0.07 lower) | - | 229 (4 RCTs) | ⨁⨁◯◯ Low^a,e^ | Medicinal plant may result in a slight reduction in nasal, eye, and throat symptoms scores (changes in mean) - Rhinorrhoea. |
| Nasal, eye, and throat symptoms scores (changes in mean) - Nasal congestion | - | SMD **0.38 lower** (0.78 lower to 0.02 higher) | - | 181 (3 RCTs) | ⨁⨁◯◯ Low^a,r^ | Medicinal plant may result in little to no difference in nasal, eye, and throat symptoms scores (changes in mean) - Nasal congestion. |
| Nasal, eye, and throat symptoms scores (changes in mean) - Nasal itchiness | - | SMD **0.28 lower** (0.68 lower to 0.11 higher) | - | 107 (2 RCTs) | ⨁⨁◯◯ Low^a,j^ | The evidence suggests that medicinal plant results in little to no difference in nasal, eye, and throat symptoms scores (changes in mean) - Nasal itchiness. |
| Nasal, eye, and throat symptoms scores (changes in mean) - Sneezing | - | SMD **0.52 lower** (0.79 lower to 0.26 lower) | - | 229 (4 RCTs) | ⨁⨁◯◯ Low^a,s^ | The evidence suggests medicinal plant results in a reduction in nasal, eye, and throat symptoms scores (changes in mean) - Sneezing. |
| Global assessment score | - | SMD **3.06 lower** (7.26 lower to 1.14 higher) | - | 236 (2 RCTs) | ⨁◯◯◯ Very low^a,m,t^ | Medicinal plant may have no effect on global assessment score but the evidence is very uncertain. |
| Responder rates (Global symptoms improvement)- pooled by rescue medication - Rescue medication | 353 per 1,000 | **385 per 1,000** (254 to 579) | **RR 1.09** (0.72 to 1.64) | 140 (2 RCTs) | ⨁⨁◯◯ Low^a,u^ | The evidence suggests that medicinal plant results in little to no difference in responder rates (Global symptoms improvement)- pooled by rescue medication - Rescue medication. |
| Responder rates (Global symptoms improvement)- pooled by rescue medication - No rescue medication | 519 per 1,000 | **820 per 1,000** (602 to 1,000) | **RR 1.58** (1.16 to 2.14) | 366 (3 RCTs) | ⨁⨁◯◯ Low^a,v^ | The evidence suggests medicinal plant results in a slight increase in responder rates (Global symptoms improvement)- pooled by rescue medication - No rescue medication. |
| Responder rates (nasal symptoms) - Rhinorrhoea | 140 per 1,000 | **790 per 1,000** (373 to 1,000) | **RR 5.66** (2.67 to 11.99) | 97 (2 RCTs) | ⨁⨁◯◯ Low^a,w^ | Medicinal plant likely results in an increase in responder rates (nasal symptoms) - Rhinorrhoea. |
| Responder rates (nasal symptoms) - Nasal congestion | 179 per 1,000 | **260 per 1,000** (14 to 1,000) | **RR 1.45** (0.08 to 27.73) | 91 (2 RCTs) | ⨁◯◯◯ Very low^a,x^ | Medicinal plant may increase/have little to no effect on responder rates (nasal symptoms) - Nasal congestion but the evidence is very uncertain. |
| Responder rates (nasal symptoms) - Nasal itchiness | 86 per 1,000 | **679 per 1,000** (251 to 1,000) | **RR 7.92** (2.93 to 21.38) | 82 (2 RCTs) | ⨁⨁◯◯ Low^a,w^ | Medicinal plant likely results in an increase in responder rates (nasal symptoms) - Nasal itchiness. |
| Responder rates (nasal symptoms) - Sneezing | 205 per 1,000 | **529 per 1,000** (131 to 1,000) | **RR 2.58** (0.64 to 10.44) | 93 (2 RCTs) | ⨁◯◯◯ Very low^a,y,z^ | Medicinal plant may increase responder rates (nasal symptoms) - Sneezing but the evidence is very uncertain. |
| Symptom duration score | The mean symptom duration score was **0** | MD **0.2 lower** (2.03 lower to 1.63 higher) | - | 59 (1 RCT) | ⨁⨁◯◯ Low^aa,k^ | The evidence suggests that medicinal plant results in no difference in symptom duration score. |
| Peak nasal inspiratory flow | The mean peak nasal inspiratory flow was **0** | MD **0.12 higher** (0.08 higher to 0.16 higher) | - | 20 (1 RCT) | ⨁⨁◯◯ Low^ab,k^ | The evidence suggests that medicinal plant results in little to no difference in peak nasal inspiratory flow. |
| RQLQ - Total score | The mean RQLQ - Total score was **0** | MD **0.46 lower** (0.84 lower to 0.07 lower) | - | 148 (3 RCTs) | ⨁⨁◯◯ Low^a,e^ | The evidence suggests medicinal plant results in a slight reduction in rQLQ - Total score. |
| RQLQ - Activity limitations | The mean RQLQ - Activity limitations was **0** | MD **0.53 lower** (1.02 lower to 0.03 lower) | - | 89 (2 RCTs) | ⨁⨁◯◯ Low^a,e^ | The evidence suggests medicinal plant results in a slight reduction in rQLQ - Activity limitations. |
| RQLQ - Sleep problems | The mean RQLQ - Sleep problems was **0** | MD **0.32 lower** (0.84 lower to 0.2 higher) | - | 89 (2 RCTs) | ⨁⨁◯◯ Low^a,j^ | The evidence suggests that medicinal plant does not reduce rQLQ - Sleep problems. |
| RQLQ - Nose symptoms | The mean RQLQ - Nose symptoms was **0** | MD **0.63 lower** (1.12 lower to 0.13 lower) | - | 89 (2 RCTs) | ⨁⨁◯◯ Low^a,j^ | The evidence suggests medicinal plant reduces rQLQ - Nose symptoms slightly. |
| RQLQ - Eye symptoms | The mean RQLQ - Eye symptoms was **0** | MD **0.54 lower** (1 lower to 0.08 lower) | - | 89 (2 RCTs) | ⨁⨁◯◯ Low^a,e^ | The evidence suggests medicinal plant reduces rQLQ - Eye symptoms slightly. |
| RQLQ - Non-nose/eye symptoms | The mean RQLQ - Non-nose/eye symptoms was **0** | MD **0.18 lower** (0.88 lower to 0.51 higher) | - | 89 (2 RCTs) | ⨁◯◯◯ Very low^a,c,y^ | Medicinal plant may have no effect on rQLQ - Non-nose/eye symptoms but the evidence is very uncertain. |
| RQLQ - Practical problem | The mean RQLQ - Practical problem was **0** | MD **0.35 lower** (0.79 lower to 0.09 higher) | - | 89 (2 RCTs) | ⨁⨁◯◯ Low^a,r^ | The evidence suggests that medicinal plant does not reduce rQLQ - Practical problem. |
| RQLQ - Emotional function | The mean RQLQ - Emotional function was **0** | MD **0.32 lower** (0.88 lower to 0.25 higher) | - | 89 (2 RCTs) | ⨁⨁◯◯ Low^a,l^ | The evidence suggests that medicinal plant does not reduce rQLQ - Emotional function. |
| Mini RQLQ - Total score | The mean mini RQLQ - Total score was **0** | MD **2.02 lower** (2.64 lower to 1.39 lower) | - | 48 (1 RCT) | ⨁⨁◯◯ Low^f,k^ | The evidence suggests medicinal plant reduces mini RQLQ - Total score. |
| Mini RQLQ - Activity limitations | The mean mini RQLQ - Activity limitations was **0** | MD **0.2 lower** (0.75 lower to 0.35 higher) | - | 70 (1 RCT) | ⨁⨁◯◯ Low^k,l^ | The evidence suggests that medicinal plant does not reduce mini RQLQ - Activity limitations. |
| Mini RQLQ - Practical problems | The mean mini RQLQ - Practical problems was **0** | MD **0.2 lower** (0.87 lower to 0.47 higher) | - | 70 (1 RCT) | ⨁⨁◯◯ Low^k,l^ | The evidence suggests that medicinal plant does not reduce mini RQLQ - Practical problems. |
| Mini RQLQ - Nose symptoms | The mean mini RQLQ - Nose symptoms was **0** | MD **0.5 lower** (1.11 lower to 0.11 higher) | - | 70 (1 RCT) | ⨁⨁◯◯ Low^k,l^ | The evidence suggests that medicinal plant does not reduce mini RQLQ - Nose symptoms. |
| Mini RQLQ - Eye symptoms | The mean mini RQLQ - Eye symptoms was **0** | MD **0.3 lower** (1.32 lower to 0.72 higher) | - | 70 (1 RCT) | ⨁⨁◯◯ Low^ac,k^ | The evidence suggests that medicinal plant does not reduce mini RQLQ - Eye symptoms. |
| Mini RQLQ - Other symptoms | The mean mini RQLQ - Other symptoms was **0** | MD **0.1 lower** (0.81 lower to 0.61 higher) | - | 70 (1 RCT) | ⨁⨁◯◯ Low^ac,k^ | The evidence suggests that medicinal plant results in little to no difference in mini RQLQ - Other symptoms. |
| Other QOL scores (unspecified) | The mean other QOL scores (unspecified) was **0** | MD **8.5 lower** (14.75 lower to 2.25 lower) | - | 33 (1 RCT) | ⨁⨁◯◯ Low^f,k^ | The evidence suggests medicinal plant reduces other QOL scores (unspecified). |
| WPAI - Working hours missed due to allergy | - | SMD **0.28 lower** (0.88 lower to 0.32 higher) | - | 43 (1 RCT) | ⨁⨁⨁◯ Moderate^l^ | Medicinal plant likely does not reduce wPAI - Working hours missed due to allergy. |
| WPAI - Effect on productivity | - | SMD **0.75 lower** (1.37 lower to 0.13 lower) | - | 43 (1 RCT) | ⨁⨁⨁◯ Moderate^ad^ | Medicinal plant probably results in little to no difference in wPAI - Effect on productivity. |
| WPAI - Effect on regular day activity | - | SMD **0.57 lower** (1.28 lower to 0.14 higher) | - | 81 (2 RCTs) | ⨁⨁◯◯ Low^j,y^ | The evidence suggests that medicinal plant results Medicinal plant may not reduce wPAI - Effect on regular day activity. |
| Medication use score | The mean medication use score was **0** | MD **0.42 lower** (1.33 lower to 0.51 higher) | - | 89 (1 RCT) | ⨁⨁⨁◯ Moderate^ac^ | Medicinal plant likely does not reduce medication use score. |
| Patients needing rescue medications | 423 per 1,000 | **558 per 1,000** (317 to 990) | **RR 1.32** (0.75 to 2.34) | 51 (1 RCT) | ⨁⨁◯◯ Low^ae,k^ | Medicinal plant may not increase patients needing rescue medications. |
| Effectiveness score | The mean effectiveness score was **0** | MD **3.9 lower** (4.35 lower to 3.45 lower) | - | 129 (1 RCT) | ⨁⨁⨁◯ Moderate^p^ | Medicinal plant probably improve effectiveness score. |
| Patient satisfaction score | The mean patient satisfaction score was **0** | MD **3.81 lower** (4.36 lower to 3.26 lower) | - | 129 (1 RCT) | ⨁⨁⨁◯ Moderate^p^ | Medicinal plant likely improves patient satisfaction score. |
| ***The risk in the intervention group** (and its 95% confidence interval) is based on the assumed risk in the comparison group and the **relative effect** of the intervention (and its 95% CI).  **CI:** confidence interval; **MD:** mean difference; **RR:** risk ratio; **SMD:** standardised mean difference | | | | | | |
| **GRADE Working Group grades of evidence** **High certainty:** we are very confident that the true effect lies close to that of the estimate of the effect. **Moderate certainty:** we are moderately confident in the effect estimate: the true effect is likely to be close to the estimate of the effect, but there is a possibility that it is substantially different. **Low certainty:** our confidence in the effect estimate is limited: the true effect may be substantially different from the estimate of the effect. **Very low certainty:** we have very little confidence in the effect estimate: the true effect is likely to be substantially different from the estimate of effect. | | | | | | |

#### Explanations

a. Most included studies had unclear risk of bias in most domains.

b. There is substantial heterogeneity, as indicated by a very high I2. There were insufficient studies for subgroup analysis.

c. Wide 95% CI that ranges from moderate reduction to moderate increase due to small cumulative sample size from small number of studies.

d. Wide 95% CI that ranges from a large reduction to a small increase in the estimate in single study of small sample size.

e. Wide 95% CI that ranges from moderate to very small reduction in estimates due to small cumulative sample size from small number of studies.

f. Small sample size which may not meet optimal information size criterion to constitute appreciable benefit.

g. There is substantial heterogeneity, as indicated by a I2 greater than 90% which led to two levels of downgrade in certainty of evidence. No major plausible sources of heterogeneity was identified after exploration.

h. Wide 95% CI that ranges from large reduction to very small reduction due to small cumulative sample size from small number of studies.

i. Wide 95% CI that ranges from large reduction to small reduction due to small cumulative sample size from small number of studies.

j. Wide 95% CI that ranges from moderate reduction to small increase due to small cumulative sample size from small number of studies.

k. Most domains had unclear risk of bias.

l. Wide 95% CI that ranges from a moderate reduction to a small increase in the estimate in single study of small sample size.

m. There is substantial heterogeneity, as indicated by a I2 greater than 90% which led to two levels of downgrade in certainty of evidence. There were insufficient studies for subgroup analysis.

n. Wide 95% CI that ranges from very large to small decrease in estimates in single study of small sample size which may not constitute appreciable benefit.

o. Wide 95% CI that ranges from small decrease to small increase in estimates small number of studies of small sample size.

p. Most domains had unclear or high risk of bias.

q. Wide 95% CI that ranges from a large reduction to moderate reduction due to small cumulative sample size from small number of studies.

r. Wide 95% CI that ranges from a moderate reduction to a very small increase due to small sample size small cumulative sample size from small number of studies.

s. Wide 95% CI that ranges from a moderate to small reduction due to small cumulative sample size from small number of studies.

t. Wide 95% CI that ranges from a large reduction to a moderate increase due to small cumulative sample size from small number of studies.

u. Wide 95% CI that ranges from moderately lower likelihood to moderately higher likelihood with small cumulative sample size from small number of studies.

v. There is substantial heterogeneity, as indicated by a high I2 of more than 50%. No major plausible sources of heterogeneity was identified after exploration.

w. Wide 95%CI due to few events in the placebo arm and small sample size which may not meet optimal information size.

x. Wide 95% CI that ranges from very unlikely to very high likelihood with small cumulative sample size from small number of studies.

y. There is substantial heterogeneity, as indicated by a high I2 of more than 50%. There were insufficient studies for subgroup analysis.

z. Wide 95% CI that ranges from moderately lower likelihood to large higher likelihood with small cumulative sample size from small number of studies

aa. Wide 95% CI that ranges from a large reduction to a moderate increase in the estimate in single study of small sample size

ab. 95% CI that ranges from a very small to small increase in estimates in single study of small sample size which may not constitute appreciable benefit.

ac. Wide 95% CI that ranges from a moderate reduction to a moderate increase in the estimate in single study of small sample size.

ad. 95% CI that ranges from moderate to small decrease in estimates in single study of small sample size which may not constitute appreciable benefit.

ae. Wide 95% CI that ranges from small lower likelihood to large higher likelihood in a single study of small sample size

**Table S2. Medicinal plant compared to oral antihistamines for Allergic Rhinitis.**

| **Medicinal plant compared to oral antihistamines for Allergic Rhinitis** | | | | | | |
| --- | --- | --- | --- | --- | --- | --- |
| **Patient or population:** Allergic Rhinitis  **Setting:** Hospital  **Intervention:** Medicinal plant  **Comparison:** Oral antihistamines | | | | | | |
| Outcomes | **Anticipated absolute effects^*^** (95% CI) | | Relative effect (95% CI) | № of participants (studies) | Certainty of the evidence (GRADE) | Comments |
|  | **Risk with oral antihistamines** | **Risk with Medicinal plant** |  |  |  |  |
| Nasal and eye symptom score (post treatment mean) - Total nasal symptoms | - | SMD **0.14 lower** (0.46 lower to 0.18 higher) | - | 149 (2 RCTs) | ⨁⨁◯◯ Low^a,b^ | Medicinal plant may not reduce nasal and eye symptom score (post treatment mean) - Total nasal symptoms. |
| Nasal and eye symptoms score (post treatment mean)- rhinorrheoa - Adults | - | SMD **0.06 lower** (0.35 lower to 0.22 higher) | - | 189 (3 RCTs) | ⨁⨁◯◯ Low^a,b^ | The evidence suggests that medicinal plant results in no difference in nasal and eye symptoms score (post treatment mean)- rhinorrheoa - Adults. |
| Nasal and eye symptoms score (post treatment mean)- rhinorrheoa - Children | - | SMD **0.58 lower** (0.87 lower to 0.29 lower) | - | 191 (1 RCT) | ⨁⨁◯◯ Low^c,d^ | The evidence suggests medicinal plant results in a slight reduction in nasal and eye symptoms score (post treatment mean)- rhinorrheoa - Children. |
| Nasal and eye symptoms score (post treatment mean)- nasal congestion | - | SMD **0.42 lower** (0.69 lower to 0.14 lower) | - | 380 (4 RCTs) | ⨁⨁◯◯ Low^a,e^ | The evidence suggests that medicinal plant results in no difference in nasal and eye symptoms score (post treatment mean)- nasal congestion. |
| Nasal and eye symptom score (post treatment mean) - Nasal itchiness | - | SMD **0.14 lower** (0.34 lower to 0.06 higher) | - | 380 (4 RCTs) | ⨁⨁◯◯ Low^a,f^ | The evidence suggests that medicinal plant does not reduce nasal and eye symptom score (post treatment mean) - Nasal itchiness. |
| Nasal and eye symptoms score (post treatment mean)- sneezing - Adults | - | SMD **0.05 lower** (0.34 lower to 0.24 higher) | - | 189 (3 RCTs) | ⨁⨁◯◯ Low^a,g^ | Medicinal plant may not reduce nasal and eye symptoms score (post treatment mean)- sneezing - Adults. |
| Nasal and eye symptoms score (post treatment mean)- sneezing - Children | - | SMD **0.63 lower** (0.93 lower to 0.34 lower) | - | 191 (1 RCT) | ⨁⨁◯◯ Low^c,d^ | The evidence suggests medicinal plant results in a slight reduction in nasal and eye symptoms score (post treatment mean)- sneezing - Children. |
| Nasal and eye symptom score (post treatment mean) - Total eye symptoms | The mean nasal and eye symptom score (post treatment mean) - Total eye symptoms was **0** | MD **0.1 lower** (0.92 lower to 0.72 higher) | - | 77 (1 RCT) | ⨁⨁◯◯ Low^c,h^ | Medicinal plant may not reduce nasal and eye symptom score (post treatment mean) - Total eye symptoms. |
| Total nasal symptom score (changes in mean) | The mean total nasal symptom score (changes in mean) was **0** | MD **0**  (1.11 lower to 1.11 higher) | - | 32 (1 RCT) | ⨁⨁◯◯ Low^c,h^ | Medicinal plant may not increase/reduce total nasal symptom score (changes in mean). |
| Nasal, eye and throat symptoms - Total nasal, eye and throat symptom (reflexive) | The mean nasal, eye and throat symptoms - Total nasal, eye and throat symptom (reflexive) was **0** | MD **0.35 lower** (1.36 lower to 0.66 higher) | - | 223 (1 RCT) | ⨁⨁◯◯ Low^c,i^ | The evidence suggests that medicinal plant does not reduce nasal, eye and throat symptoms - Total nasal, eye and throat symptom (reflexive). |
| Nasal, eye and throat symptoms - Total nasal, eye and throat symptom (instantaneous) | The mean nasal, eye and throat symptoms - Total nasal, eye and throat symptom (instantaneous) was **0** | MD **0**  (2.92 lower to 2.92 higher) | - | 223 (1 RCT) | ⨁⨁◯◯ Low^c,j^ | The evidence suggests that medicinal plant does not increase/reduce nasal, eye and throat symptoms - Total nasal, eye and throat symptom (instantaneous). |
| Global assessment score | The mean global assessment score was **0** SD | MD **0.02 SD lower** (0.69 lower to 0.65 higher) | - | 77 (1 RCT) | ⨁⨁◯◯ Low^c,h^ | The evidence suggests that medicinal plant does not reduce global assessment score. |
| Responder rates (global symptoms) | 47 per 1,000 | **328 per 1,000** (134 to 802) | **RR 7.01** (2.86 to 17.16) | 220 (1 RCT) | ⨁⨁⨁◯ Moderate^c^ | Medicinal plant likely results in an increase in responder rates (global symptoms). |
| RCAT | The mean RCAT was **0** | MD **1.14 lower** (3.23 lower to 0.95 higher) | - | 77 (1 RCT) | ⨁⨁◯◯ Low^c,k^ | The evidence suggests that medicinal plant does not reduce rCAT. |
| Throat symptoms | The mean throat symptoms was **0** SD | MD **0.01 SD higher** (0.74 lower to 0.76 higher) | - | 77 (1 RCT) | ⨁⨁◯◯ Low^c,h^ | The evidence suggests that medicinal plant does not increase throat symptoms. |
| Ear symptoms | The mean ear symptoms was **0** | MD **0**  (0.76 lower to 0.76 higher) | - | 77 (1 RCT) | ⨁⨁◯◯ Low^c,h^ | The evidence suggests that medicinal plant does not increase/reduce ear symptoms. |
| Post nasal drip | The mean post nasal drip was **0** | MD **0.07 lower** (0.8 lower to 0.66 higher) | - | 77 (1 RCT) | ⨁⨁◯◯ Low^c,h^ | Medicinal plant may not reduce post nasal drip. |
| Headache | The mean headache was **0** | MD **0.26 higher** (0.42 lower to 0.94 higher) | - | 77 (1 RCT) | ⨁⨁◯◯ Low^c,l^ | Medicinal plant may not improve headache. |
| Mental function | The mean mental function was **0** | MD **0.17 higher** (0.59 lower to 0.93 higher) | - | 77 (1 RCT) | ⨁⨁◯◯ Low^c,h^ | The evidence suggests that medicinal plant does not improve mental function. |
| Cough | The mean cough was **0** | MD **0.01 higher** (0.56 lower to 0.58 higher) | - | 77 (1 RCT) | ⨁⨁◯◯ Low^c,h^ | The evidence suggests that medicinal plant does not improve cough. |
| Measurements on cross sectional area of the nasal cavity - Minimal cross section area of right nose | The mean measurements on cross sectional area of the nasal cavity - Minimal cross section area of right nose was **0** | MD **0.05 higher** (0.02 lower to 0.12 higher) | - | 72 (1 RCT) | ⨁⨁⨁◯ Moderate^m^ | Medicinal plant likely results in little to no difference in measurements on cross sectional area of the nasal cavity - Minimal cross section area of right nose. |
| Measurements on cross sectional area of the nasal cavity - Minimal cross section area of left nose | The mean measurements on cross sectional area of the nasal cavity - Minimal cross section area of left nose was **0** | MD **0.04 higher** (0.01 lower to 0.09 higher) | - | 72 (1 RCT) | ⨁⨁⨁◯ Moderate^m^ | Medicinal plant likely results in little to no difference in measurements on cross sectional area of the nasal cavity - Minimal cross section area of left nose. |
| Measurements on cross sectional area of the nasal cavity - Volume estimates of the right nasal cavity | The mean measurements on cross sectional area of the nasal cavity - Volume estimates of the right nasal cavity was **0** | MD **0.75 higher** (0.14 higher to 1.36 higher) | - | 72 (1 RCT) | ⨁⨁◯◯ Low^m,n^ | The evidence suggests that medicinal plant results in little to no difference in measurements on cross sectional area of the nasal cavity - Volume estimates of the right nasal cavity. |
| Measurements on cross sectional area of the nasal cavity - Volume estimates of the left nasal cavity | The mean measurements on cross sectional area of the nasal cavity - Volume estimates of the left nasal cavity was **0** | MD **0.58 higher** (0.08 higher to 1.08 higher) | - | 72 (1 RCT) | ⨁⨁◯◯ Low^m,o^ | The evidence suggests that medicinal plant results in little to no difference in measurements on cross sectional area of the nasal cavity - Volume estimates of the left nasal cavity. |
| Measurements on cross sectional area of the nasal cavity - Distance from the nostril of right nose | The mean measurements on cross sectional area of the nasal cavity - Distance from the nostril of right nose was **0** | MD **0.03 higher** (0.1 lower to 0.16 higher) | - | 72 (1 RCT) | ⨁⨁⨁◯ Moderate^m^ | Medicinal plant likely does not increase measurements on cross sectional area of the nasal cavity - Distance from the nostril of right nose. |
| Measurements on cross sectional area of the nasal cavity - Distance from the nostril of left nose | The mean measurements on cross sectional area of the nasal cavity - Distance from the nostril of left nose was **0** | MD **0.19 lower** (0.34 lower to 0.04 lower) | - | 72 (1 RCT) | ⨁⨁◯◯ Low^m,p^ | Medicinal plant probably results in little to no difference in measurements on cross sectional area of the nasal cavity - Distance from the nostril of left nose. |
| Physical examination of nasal cavity - Inferior turbinate swelling score | The mean physical examination of nasal cavity - Inferior turbinate swelling score was **0** | MD **0.43 lower** (0.87 lower to 0 ) | - | 191 (1 RCT) | ⨁◯◯◯ Very low^c,m,q^ | Medicinal plant may have little to no effect on physical examination of nasal cavity - Inferior turbinate swelling score but the evidence is very uncertain. |
| Physical examination of nasal cavity - Nasal mucosa oedema score | The mean physical examination of nasal cavity - Nasal mucosa oedema score was **0** | MD **0.59 lower** (0.72 lower to 0.46 lower) | - | 191 (1 RCT) | ⨁⨁◯◯ Low^c,m^ | Medicinal plant may result in a slight reduction in physical examination of nasal cavity - Nasal mucosa oedema score. |
| Physical examination of nasal cavity - Nasal mucosa swelling score | The mean physical examination of nasal cavity - Nasal mucosa swelling score was **0** | MD **0.15 lower** (0.26 lower to 0.04 lower) | - | 191 (1 RCT) | ⨁◯◯◯ Very low^c,m,p^ | The evidence suggests that medicinal plant results in little to no difference in physical examination of nasal cavity - Nasal mucosa swelling score. |
| Peak nasal inspiratory flow | The mean peak nasal inspiratory flow was **0** | MD **12 lower** (14.08 lower to 9.92 lower) | - | 32 (1 RCT) | ⨁⨁⨁◯ Moderate^c^ | Medicinal plant likely results in a reduction in peak nasal inspiratory flow. |
| RQLQ - Total | The mean RQLQ - Total was **0** | MD **0.1 lower** (0.56 lower to 0.36 higher) | - | 72 (1 RCT) | ⨁⨁⨁◯ Moderate^r^ | Medicinal plant likely does not reduce rQLQ - Total. |
| RQLQ - Activity limitations | The mean RQLQ - Activity limitations was **0** | MD **0.4 lower** (1.01 lower to 0.21 higher) | - | 72 (1 RCT) | ⨁⨁⨁◯ Moderate^r^ | Medicinal plant likely does not reduce rQLQ - Activity limitations. |
| RQLQ - Sleep problems | The mean RQLQ - Sleep problems was **0** | MD **0.05 lower** (0.62 lower to 0.52 higher) | - | 72 (1 RCT) | ⨁⨁⨁◯ Moderate^h^ | Medicinal plant likely does not reduce rQLQ - Sleep problems. |
| RQLQ - Nose symptoms | The mean RQLQ - Nose symptoms was **0** | MD **0.29 lower** (0.91 lower to 0.33 higher) | - | 72 (1 RCT) | ⨁⨁⨁◯ Moderate^r^ | Medicinal plant likely does not reduce rQLQ - Nose symptoms. |
| RQLQ - Eye symptoms | The mean RQLQ - Eye symptoms was **0** | MD **0.05 higher** (0.51 lower to 0.61 higher) | - | 72 (1 RCT) | ⨁⨁⨁◯ Moderate^h^ | Medicinal plant likely does not reduce rQLQ - Eye symptoms. |
| RQLQ - Non-nose/eye symptoms | The mean RQLQ - Non-nose/eye symptoms was **0** | MD **0.07 lower** (0.61 lower to 0.47 higher) | - | 72 (1 RCT) | ⨁⨁⨁◯ Moderate^r^ | Medicinal plant likely does not reduce rQLQ - Non-nose/eye symptoms. |
| RQLQ - Practical problem | The mean RQLQ - Practical problem was **0** | MD **0.02 lower** (0.61 lower to 0.57 higher) | - | 72 (1 RCT) | ⨁⨁⨁◯ Moderate^h^ | Medicinal plant likely does not reduce rQLQ - Practical problem. |
| RQLQ - Emotional function | The mean RQLQ - Emotional function was **0** | MD **0.02 lower** (0.48 lower to 0.44 higher) | - | 72 (1 RCT) | ⨁⨁⨁◯ Moderate^s^ | Medicinal plant likely does not reduce rQLQ - Emotional function. |
| Other QOL scores (unspecified) | The mean other QOL scores (unspecified) was **0** | MD **0.2 higher** (0.44 lower to 0.84 higher) | - | 77 (1 RCT) | ⨁⨁◯◯ Low^c,l^ | The evidence suggests that medicinal plant does not increase other QOL scores (unspecified). |
| ***The risk in the intervention group** (and its 95% confidence interval) is based on the assumed risk in the comparison group and the **relative effect** of the intervention (and its 95% CI).  **CI:** confidence interval; **MD:** mean difference; **RR:** risk ratio; **SMD:** standardised mean difference | | | | | | |
| **GRADE Working Group grades of evidence** **High certainty:** we are very confident that the true effect lies close to that of the estimate of the effect. **Moderate certainty:** we are moderately confident in the effect estimate: the true effect is likely to be close to the estimate of the effect, but there is a possibility that it is substantially different. **Low certainty:** our confidence in the effect estimate is limited: the true effect may be substantially different from the estimate of the effect. **Very low certainty:** we have very little confidence in the effect estimate: the true effect is likely to be substantially different from the estimate of effect. | | | | | | |

#### Explanations

a. Most included studies had unclear risk of bias in most domains.

b. Wide 95% CI that ranges from a small reduction to a small increase in estimates due to small cumulative sample size from a small number of studies.

c. Most domains had unclear risk of bias.

d. Wide 95% CI that ranges from moderate to small reduction in estimates from a single study of small sample size.

e. Wide 95% CI that ranges from a moderate to small decrease in estimates due to small cumulative sample size from a small number of studies.

f. Wide 95% CI that ranges from a small reduction to a very small increase in estimates due to small cumulative sample size from a small number of studies.

g. Wide 95% CI that ranges from a small reduction to a small increase in estimates due to small cumulative sample size from a small number of studies.

h. Wide 95% CI that ranges from a moderate reduction to a moderate increase in estimates from a single study of small sample size.

i. Wide 95% CI that ranges from a moderate reduction to a moderate increase in estimates from a single study.

j. Wide 95% CI that ranges from a large reduction to a large increase in estimates from a single study.

k. Wide 95% CI that ranges from a large reduction to a moderate increase in estimates from a single study of small sample size.

l. Wide 95% CI that ranges from a small reduction to a moderate increase in estimates from a single study of small sample size.

m. The outcome measured was not an established clinical examination routinely recommended for severity grading in clinical practice guidelines.

n. Wide 95% CI that ranges from a small to a moderate increase in estimates from a single study of small sample size.

o. Wide 95% CI that ranges from a very small to a moderate increase in estimates in a single study of small sample size.

p. Wide 95% CI that ranges from small to very small reduction in estimates from a single study of small sample size.

q. Wide 95% CI that ranges from a small decrease to no differences in estimates from a single study of small sample size.

r. Wide 95% CI that ranges from a moderate reduction to a small increase in estimates from a single study of small sample size.

s. Wide 95% CI that ranges from a small reduction to a small increase in estimates from a single study of small sample size.

**Table S3. Medicinal plant compared to intranasal corticosteroids for Allergic Rhinitis.**

| **Medicinal plant compared to intranasal corticosteroids for Allergic Rhinitis** | | | | | | |
| --- | --- | --- | --- | --- | --- | --- |
| **Patient or population:** Allergic Rhinitis  **Setting:** Hospital  **Intervention:** Medicinal plant  **Comparison: I**ntranasal corticosteroids | | | | | | |
| Outcomes | **Anticipated absolute effects^*^** (95% CI) | | Relative effect (95% CI) | № of participants (studies) | Certainty of the evidence (GRADE) | Comments |
|  | **Risk with inhaled corticosteroids** | **Risk with Medicinal plant** |  |  |  |  |
| Total nasal symptom score | The mean total nasal symptom score was **0** | MD **0.29 higher** (0.03 higher to 0.54 higher) | - | 21 (1 RCT) | ⨁⨁◯◯ Low^a,b^ | The evidence suggests medicinal plant results in a slight increase in total nasal symptom score. |
| ***The risk in the intervention group** (and its 95% confidence interval) is based on the assumed risk in the comparison group and the **relative effect** of the intervention (and its 95% CI).  **CI:** confidence interval; **MD:** mean difference | | | | | | |
| **GRADE Working Group grades of evidence** **High certainty:** we are very confident that the true effect lies close to that of the estimate of the effect. **Moderate certainty:** we are moderately confident in the effect estimate: the true effect is likely to be close to the estimate of the effect, but there is a possibility that it is substantially different. **Low certainty:** our confidence in the effect estimate is limited: the true effect may be substantially different from the estimate of the effect. **Very low certainty:** we have very little confidence in the effect estimate: the true effect is likely to be substantially different from the estimate of effect. | | | | | | |

#### Explanations

a. Most domains had unclear risk of bias.

b. Wide 95% CI that ranges from a very small reduction to moderate increase in the estimates in a single study of small sample size.

**Table S4. Medicinal plant (as add-on) compared to standard treatment for Allergic Rhinitis**

| **Medicinal plant (as add-on) compared to standard treatment for Allergic Rhinitis** | | | | | | |
| --- | --- | --- | --- | --- | --- | --- |
| **Patient or population:** Allergic Rhinitis  **Setting:** Hospital or other unspecified setting  **Intervention:** Medicinal plant (as add-on to standard treatment)  **Comparison:** Standard treatment | | | | | | |
| Outcomes | **Anticipated absolute effects^*^** (95% CI) | | Relative effect (95% CI) | № of participants (studies) | Certainty of the evidence (GRADE) | Comments |
|  | **Risk with standard treatment** | **Risk with Medicinal plant (as add-on)** |  |  |  |  |
| Nasal and eye symptom score (post intervention mean) - Total nasal symptom score | The mean nasal and eye symptom score (post intervention mean) - Total nasal symptom score was **0** | MD **1 lower** (3.46 lower to 1.46 higher) | - | 16 (1 RCT) | ⨁⨁◯◯ Low^a,b^ | Medicinal plant (as add-on) may not reduce nasal and eye symptom score (post intervention mean) - Total nasal symptom score. |
| Nasal and eye symptom score (post intervention mean) - Rhinorrhoea | The mean nasal and eye symptom score (post intervention mean) - Rhinorrhoea was **0** | MD **0**  (0.74 lower to 0.74 higher) | - | 16 (1 RCT) | ⨁⨁◯◯ Low^a,c^ | The evidence suggests that medicinal plant (as add-on) does not increase/reduce nasal and eye symptom score (post intervention mean) - Rhinorrhoea. |
| Nasal and eye symptom score (post intervention mean) - Nasal congestion | The mean nasal and eye symptom score (post intervention mean) - Nasal congestion was **0** | MD **0.5 lower** (1.55 lower to 0.55 higher) | - | 16 (1 RCT) | ⨁⨁◯◯ Low^a,c^ | Medicinal plant (as add-on) may not reduce nasal and eye symptom score (post intervention mean) - Nasal congestion. |
| Nasal and eye symptom score (post intervention mean) - Nasal itchiness | The mean nasal and eye symptom score (post intervention mean) - Nasal itchiness was **0** | MD **0.5 lower** (1.24 lower to 0.24 higher) | - | 16 (1 RCT) | ⨁⨁◯◯ Low^a,d^ | The evidence suggests that medicinal plant (as add-on) does not reduce nasal and eye symptom score (post intervention mean) - Nasal itchiness. |
| Nasal and eye symptom score (post intervention mean) - Sneezing | The mean nasal and eye symptom score (post intervention mean) - Sneezing was **0** | MD **0.13 higher** (0.61 lower to 0.87 higher) | - | 16 (1 RCT) | ⨁⨁◯◯ Low^a,c^ | Medicinal plant (as add-on) may not increase nasal and eye symptom score (post intervention mean) - Sneezing. |
| Nasal and eye symptom score (post intervention mean) - Total eye symptom score | The mean nasal and eye symptom score (post intervention mean) - Total eye symptom score was **0** | MD **0.75 lower** (2 lower to 0.5 higher) | - | 16 (1 RCT) | ⨁⨁◯◯ Low^a,b^ | The evidence suggests that medicinal plant (as add-on) does not reduce nasal and eye symptom score (post intervention mean) - Total eye symptom score. |
| Nasal and eye symptom score (post intervention mean) - Watery eyes | The mean nasal and eye symptom score (post intervention mean) - Watery eyes was **0** | MD **0.12 lower** (0.76 lower to 0.52 higher) | - | 16 (1 RCT) | ⨁⨁◯◯ Low^a,c^ | The evidence suggests that medicinal plant (as add-on) does not reduce nasal and eye symptom score (post intervention mean) - Watery eyes. |
| Nasal and eye symptom score (post intervention mean) - Itchy eyes | The mean nasal and eye symptom score (post intervention mean) - Itchy eyes was **0** | MD **0.5 lower** (1.14 lower to 0.14 higher) | - | 16 (1 RCT) | ⨁⨁◯◯ Low^a,e^ | The evidence suggests that medicinal plant (as add-on) does not reduce nasal and eye symptom score (post intervention mean) - Itchy eyes. |
| Nasal and eye symptom score (post intervention mean) - Red eyes | The mean nasal and eye symptom score (post intervention mean) - Red eyes was **0** | MD **0.13 lower** (0.37 lower to 0.11 higher) | - | 16 (1 RCT) | ⨁⨁◯◯ Low^a,d^ | The evidence suggests that medicinal plant (as add-on) does not reduce nasal and eye symptom score (post intervention mean) - Red eyes. |
| Nasal symptom score (changes in mean) - Total score | The mean nasal symptom score (changes in mean) - Total score was **0** | MD **0.31 higher** (0.05 higher to 0.57 higher) | - | 21 (1 RCT) | ⨁⨁◯◯ Low^a,f^ | The evidence suggests medicinal plant (as add-on) results in a slight worsening in nasal symptom score (changes in mean) - Total score. |
| SNOT-22 - oral | The mean SNOT-22 was **0** | MD **15.58 lower** (38.9 lower to 7.74 higher) | - | 63 (2 RCTs) | ⨁◯◯◯ Very low^j,k^ | The evidence is very uncertain. Medicinal plant (as add-on) may not reduce SNOT-22 scores when given orally when added on to conventional therapy. |
| SNOT-22 - intranasal | The mean SNOT-22 was **0** | MD **7.47 lower** (10.75 lower to 4.18 lower) | - | 124 (4 RCTs) | ⨁⨁⨁◯ Moderate^l^ | Medicinal plant (as add-on) likely reduces sNOT-22 - Intranasal. |
| Lund–McKay score | The mean lund–McKay score was **0** | MD **1.88 lower** (2.44 lower to 1.32 lower) | - | 65 (1 RCT) | ⨁⨁⨁⨁ High | Medicinal plant (as add-on) results in a reduction in lund–McKay score. |
| Modified Lund Kennedy score | The mean modified Lund Kennedy score was **0** | MD **1.89 lower** (2.4 lower to 1.38 lower) | - | 65 (1 RCT) | ⨁⨁⨁⨁ High | Medicinal plant (as add-on) results in a reduction in modified Lund Kennedy score. |
| Nasal airway resistance | The mean nasal airway resistance was **0** | MD **0.05 lower** (0.29 lower to 0.19 higher) | - | 16 (1 RCT) | ⨁⨁⨁◯ Moderate^a^ | Medicinal plant (as add-on) likely does not reduce nasal airway resistance. |
| ***The risk in the intervention group** (and its 95% confidence interval) is based on the assumed risk in the comparison group and the **relative effect** of the intervention (and its 95% CI).  **CI:** confidence interval; **MD:** mean difference | | | | | | |
| **GRADE Working Group grades of evidence** **High certainty:** we are very confident that the true effect lies close to that of the estimate of the effect. **Moderate certainty:** we are moderately confident in the effect estimate: the true effect is likely to be close to the estimate of the effect, but there is a possibility that it is substantially different. **Low certainty:** our confidence in the effect estimate is limited: the true effect may be substantially different from the estimate of the effect. **Very low certainty:** we have very little confidence in the effect estimate: the true effect is likely to be substantially different from the estimate of effect. | | | | | | |

#### Explanations

a. Most domains had unclear risk of bias.

b. Wide 95% CI that ranges from a large reduction to a moderate increase in estimates in a single study of small sample size.

c. Wide 95% CI that ranges from a moderate reduction to a moderate increase in estimates in a single study of small sample size.

d. Wide 95% CI that ranges from a moderate reduction to a small increase in estimates in a single study of small sample size.

e. Wide 95% CI that ranges from a large reduction to a small increase in estimates in a single study of small sample size.

f. Wide 95% CI that ranges from a very small to moderate increase in estimates in a single study of small sample size

g. Most of the included studies had unclear risk of bias.

h. There is substantial heterogeneity, as indicated by a high I2 of more than 50%. No major plausible sources of heterogeneity was identified after exploration.

i. Wide 95% CI that ranges from a very large to moderate reduction in estimates with small cumulative sample size from a small number of studies.

j. There is substantial heterogeneity, as indicated by a high I2 of more than 50%. There were insufficient studies to further explore additional plausible reasons for heterogeneity.

k. Very wide 95% CI that ranges from a very large decrease to large increase in estimates due to small cumulative sample size from a small number of studies.

l. Wide 95% CI that ranges from a large to moderate decrease in estimates due to small cumulative sample size from a small number of studies.
