# Supplementary material for: Medicinal plants for allergic rhinitis: A systematic review and meta-analysis
Source: PLoS One. 2024 Apr 11;19(4):e0297839. doi: 10.1371/journal.pone.0297839 (PMC11008904; doi:10.1371/journal.pone.0297839)
Supplement: S11 Appendix — (PDF) [file pone.0297839.s011.pdf]

Appendix S11. Reporting quality of herbal preparations in randomised controlled trials

Author, Year	Herbal medicinal product name			Characteristics of the herbal product		
	Latin binomial name together with botanical authority and family name for each herbal ingredient	Poduct name (i.e., brand name) or the extract name (e.g., EGb-761) and the name of the manufacturer of the product.	Whether the product used is authorised (licensed, registered) in the country in which the study was conducted.	Part(s) of plant used	Type of product used (e.g., raw [fresh or dry], extract).	Type and concentration of extraction solvent used
Achararit, 2023	Yes	Yes	No	No	Yes	No
Ariaee, 2021	No	No	No	No	No	Yes
Arpornchayanon, 2019	Yes	No	No	No	No	No
Atar, 2022	Yes	Yes	No	No	No	No
Badar, 2005	Yes	Yes	No	Yes	Yes	No
Bakhshaee, 2017	Yes	Yes	No	Yes	Yes	No
Bernstein, 2002	No	No	No	Yes	Yes	No
Derakhshan, 2019	No	Yes	No	Yes	Yes	Yes
Cingi, 2008	No	No	No	Yes	Yes	N/A
Gray, 2004	No	Yes	No	No	No	No
Hajiheydari, 2017	Yes	Yes	No	No	Yes	Yes
Jung, 2011	No	Yes	No	No	No	No
Jung, 2021	Yes	No	No	No	No	No
Lee, 2004	Yes	No	Yes	Yes	Yes	Yes
Matkovic, 2010	No	No	No	No	Yes	No
Mittman, 1990	No	No	No	No	Yes	No
Rezaeian, 2018	No	No	No	No	No	No
Schapowal, 2002	No	Yes	No	No	Yes	No
Schapowal, 2004	No	Yes	No	Yes	Yes	No
Schapowal, 2005	No	Yes	No	Yes	Yes	No
Steels, 2019	No	Yes	No	Yes	Yes	No
Takano, 2004	No	N/A	No	Yes	Yes	Yes
Walanj, 2014	No	Yes	No	Yes	Yes	No
Wilson, 2010	Yes	Yes	No	Yes	Yes	No
Wu, 2009	No	Yes	No	No	Yes	No
Yamprasert, 2020	Yes	N/A	No	Yes	Yes	Yes
Yonekura, 2011	No	No	No	No	No	No
Yoshimura, 2007	Yes	N/A	No	Yes	Yes	Yes
Yusin, 2021	No	Yes	No	Yes	Yes	No

Author, Year	Dosage regimen and quantitative description				Qualitative testing
	Method of authentication of raw material (i.e., how done and by whom) and the lot number of the raw material. State if a voucher specimen (i.e., retention sample) was retained and, if so, where it is kept or deposited, and the reference number	The dosage of the product, the duration of administration, and how these were determined.	The content (e.g., as weight, concentration; may be given as range where appropriate) of all quantified herbal product constituents, both native and added, per dosage unit form	For standardised products, the quantity of active/marker constituents per dosage unit form.	Chemical fingerprint and methods used
Achararit, 2023	N/A	No	Yes	Yes	No
Ariaee, 2021	N/A	No	Yes	N/A	No
Arpornchayanon, 2019	No	No	No	N/A	Yes
Atar, 2022	N/A	No	No	No	No
Badar, 2005	N/A	No	No	Yes	No
Bakhshaee, 2017	N/A	No	No	N/A	No
Bernstein, 2002	No	No	No	Yes	No
Derakhshan, 2019	No	Yes	Yes	Yes	No
Cingi, 2008	No	No	Yes	N/A	No
Gray, 2004	N/A	No	No	N/A	No
Hajiheydari, 2017	Yes	Yes	Yes	Yes	No
Jung, 2011	N/A	No	No	No	No
Jung, 2021	N/A	No	No	No	No
Lee, 2004	N/A	No	No	Yes	Yes
Matkovic, 2010	N/A	No	No	No	No
Mittman, 1990	N/A	No	Yes	Yes	No
Rezaeian, 2018	N/A	No	No	No	No
Schapowal, 2002	N/A	No	No	Yes	No
Schapowal, 2004	No	No	No	Yes	No
Schapowal, 2005	No	No	No	Yes	No
Steels, 2019	N/A	No	No	Yes	No
Takano, 2004	No	No	No	N/A	Yes
Walanj, 2014	No	No	No	Yes	No
Wilson, 2010	N/A	No	No	No	No
Wu, 2009	No	No	No	N/A	No
Yamprasert, 2020	Yes	Yes	No	N/A	Yes
Yonekura, 2011	No	No	No	N/A	No
Yoshimura, 2007	No	No	No	N/A	No
Yusin, 2021	N/A	No	No	Yes	No

Author, Year	Qualitative testing		Placebo/control group	Practitioner
	**Description of any special testing/purity testing (e.g., heavy metal or other contaminant testing) undertaken, which unwanted components were removed and how (i.e., methods)**	**Standardisation: what to standardize (e.g., which chemical components of the product) and how (e.g., chemical processes or biological/functional measures of activity).**	**The rationale for the type of control/placebo used.**	**A description of the practitioners (e.g., training and practice experience) that are a part of the intervention**
Achararit, 2023	No	No	Yes	No
Ariaee, 2021	No	N/A	No	No
Arpornchayanon, 2019	No	N/A	No	No
Atar, 2022	No	No	No	No
Badar, 2005	No	No	Yes	No
Bakhshaee, 2017	No	N/A	Yes	No
Bernstein, 2002	No	Yes	Yes	No
Derakhshan, 2019	No	Yes	No	No
Cingi, 2008	Yes	N/A	Yes	No
Gray, 2004	No	N/A	Yes	No
Hajiheydari, 2017	No	Yes	Yes	No
Jung, 2011	No	No	No	No
Jung, 2021	No	No	No	No
Lee, 2004	No	Yes	Yes	No
Matkovic, 2010	No	N/A	Yes	No
Mittman, 1990	No	No	Yes	No
Rezaeian, 2018	No	N/A	Yes	No
Schapowal, 2002	No	No	No	No
Schapowal, 2004	No	No	No	No
Schapowal, 2005	No	No	No	No
Steels, 2019	No	No	Yes	No
Takano, 2004	No	N/A	Yes	No
Walanj, 2014	Yes	No	Yes	No
Wilson, 2010	No	No	Yes	No
Wu, 2009	No	No	No	No
Yamprasert, 2020	Yes	N/A	Yes	No
Yonekura, 2011	No	N/A	No	No
Yoshimura, 2007	No	N/A	Yes	No
Yusin, 2021	No	No	Yes	No
