# Supplementary material for: Medicinal plants for allergic rhinitis: A systematic review and meta-analysis
Source: PLoS One. 2024 Apr 11;19(4):e0297839. doi: 10.1371/journal.pone.0297839 (PMC11008904; doi:10.1371/journal.pone.0297839)
Supplement: Attachment — Submitted filename: Submit Recommendation.docx [file pone.0297839.s013.docx]

This systematic review and meta-analysis focuses on the effectiveness and safety of individual medicinal plants in allergic rhinitis,trying to emphasize medicinal plants as a significant complementary and alternative treatment option based on its reasonable clinical effectiveness, good patient compliance and minimal adverse reactions.However,the manuscript lacks originality and offers no novel insights.Therefore a rejection of the manuscript is suggested for the following reasons:

1.Part of the data needs to be verified.The conclusion states, "From the initial 1,523 articles identified, 39 articles were included, comprising 29 published randomized controlled trials. We identified 10 unpublished or ongoing registered trials." However, Figure 1 and the abstract mentioned only 29 cases.

2.Although there's no length restriction for manuscripts writing, concise presentation style and discussion based on the findings are encouraged.Parts of the manuscript are repetitive and redundant, particularly in the sections on Search Strategy, Study Selection, and Data Collection.

3.The criteria for inclusion and exclusion of literature are crucial for the outcomes of the meta-analysis and the overall quality of the article. I recommend adding these criteria in the main text rather than referring to Appendix S1.

4.Due to different language barrier in relation to literature availability in journals published in Chinese,this study has not thoroughly covered Chinese literature. Therefore it is suggested to supplement these data.

5.The study involved a variety of medicinal plants and their components. This diversity could lead to a significant bias in the research outcomes, making it challenging to draw reliable and unified conclusions. The variation in medicinal plants ingredients across the studies should be addressed to ensure the validity and applicability of the findings

6.Various measures and scales were used in the study, particularly for overall assessment，general and specific nasal symptoms, some contradictory results (as seen in Figures 4, 5, and 8) were reported. Although explanations have been provided for these discrepancies, it seems that the results were not so reliable.
